# Prevalence estimates of genital *Chlamydia trachomatis* infection in Belgium: results from two cross-sectional studies

**DOI:** 10.1186/s12879-021-06646-y

**Published:** 2021-09-14

**Authors:** Natalie Fischer, Ilse Peeters, Sofieke Klamer, Marion Montourcy, Vicky Cuylaerts, Dominique Van Beckhoven, Irith De Baetselier, Johan Van der Heyden, Wim Vanden Berghe

**Affiliations:** 1 Service Epidemiology of Infectious Diseases, Sciensano, Rue Ernest Blerot 1, 1070 Anderlecht, Belgium; 2 European Programme for Public Health Microbiology (EUPHEM), European Centre for Disease Prevention and Control (ECDC), Gustav III:s Boulevard 40, 169 73 Solna, Sweden; 3 Department of Clinical Sciences, National Reference Centre for Sexually Transmitted Infections, Clinical Reference Laboratory, Institute of Tropical Medicine, Nationalestraat 155, 2000 Antwerp, Belgium; 4 Service Lifestyle and Chronic Diseases, Sciensano, Rue Ernest Blerot 1, 1070 Anderlecht, Belgium

**Keywords:** *Chlamydia trachomatis*, Prevalence, Cross-sectional study, Belgium, Sexually transmitted infections

## Abstract

**Background:**

*Chlamydia trachomatis* (chlamydia) is the most diagnosed sexually transmitted infection in Belgium. Screening programs focus on young women, due to the implications of chronic asymptomatic infections for reproductive health. Thereby, the frequency of infections in men and older adults is underestimated. This study aimed to estimate the point-prevalence of chlamydia in the broader Belgian population, to inform evidence-based prevention and control strategies.

**Methods:**

We conducted two cross-sectional prevalence studies of chlamydia infection in the population of Belgium aged 16–59 years, 2018–2020. In the CT1 study 12,000 representative individuals were randomly selected from the national register and invited by letter to collect a urine sample at home. The CT2 study used urine samples collected through the Belgian Health Examination Survey. Molecular detection of chlamydia DNA was performed using Xpert^®^ or Abbott Real-Time CT/NG assays. Weighted estimated prevalence and 95% confidence interval (CI) was calculated per gender and age groups of 16/18–29, 30–44 and 45–59 years, relative to the general Belgian population. Data collected on sociodemographic variables and sexual behavior were used to identify potential risk factors for chlamydia infection through calculation of the odds ratio (OR).

**Results:**

The population-wide weighted estimated prevalence was 1.54% (95% CI 0.78–3) in CT1 and 1.76% (95% CI 0.63–4) in CT2. We observed no statistically significant difference between men and women or age groups. Civil relationship status (OR = 14.1 (95% CI 1.78–112), p < 0.01), sexual intercourse with a casual partner (OR = 6.31 (95% CI 1.66–24.1), p < 0.01) and > 3 sexual partners in the last 12 months (OR = 4.53 (95% CI 1.10–18.6), p = 0.02) were associated with higher relative risk for chlamydia infection.

**Conclusion:**

Nationwide prevalence studies are relevant to assess the distribution of chlamydia and inform public health actions. The overall low prevalence and heterogeneous distribution of chlamydia in the general Belgian population needs to be considered for future strategies and potential harm of testing and treating asymptomatic individuals need to be taken into account. Effective case management should include appropriate treatment of symptomatic patients and partner notification, and prevention strategies should encourage behaviors such as condom use.

**Supplementary Information:**

The online version contains supplementary material available at 10.1186/s12879-021-06646-y.

## Background

*Chlamydia trachomatis *(*C. trachomatis,* commonly known as chlamydia) is an obligate intracellular gram-negative bacterium, able to cause a variety of infections in humans [1]. Chlamydia is the most diagnosed sexually transmittable infection (STI) in the Western world, also in Belgium [2]. Genital infection with chlamydia predominantly affects the lower urogenital tract and can cause inflammation of the vagina, cervix, urethra or penis [3]. However, most genital infections are asymptomatic and self-limiting, and therefore are commonly underdiagnosed. Especially in men, only around 10% develop urethritis with urethral discharge, and a minority may present with epididymitis [4]. In women, untreated infections may lead to complications such as pelvic inflammatory disease (PID), chronic pelvic pain, ectopic pregnancies and infertility [5]. The risk to develop PID which is attributable to untreated chlamydia infection is estimated between 10–30% [5, 6]. National efforts for early detection and treatment through screening programs are aimed to reduce transmission and the burden of disease with regards to reproductive health [7]. However, the relevance and harm-benefit ratio of active case finding and treatment of asymptomatic infections has recently been under debate [8].

In 2018, the average number of total reported chlamydia cases across 26 European countries was 146 per 100,000 inhabitants [9]. The prevalence of chlamydia in Europe was recently estimated as 2.7% (95% CI 1.9–3.6) in a meta-analysis including nine European countries, with no significant difference between men and women [10]. Nevertheless, national burden of disease estimates are largely dependent on the extend of the surveillance systems and young women are prioritized by testing policies across countries. Moreover, the true prevalence of chlamydia in Europe is likely to be higher partly due to a high proportion of asymptomatic infections that remain undetected [11].

In Belgium, routine screening is recommended for sexually active adolescents and young adults between 15–29 years of age and for specific risk groups such as men who have sex with men [12]. Following a positive nucleic acid amplification test, treatment with doxycycline or azithromycin is initiated and partner testing and treatment is recommended [13]. The national laboratory sentinel-network reported a 10% increase of cases from 69 per 100,000 inhabitants in 2017 to 77 per 100,000 inhabitants in 2019. For the same period, the number of reimbursed tests for chlamydia also increased. Infections are more commonly reported among women, where the number of cases is highest in the age group of 15–30 years [2]. In men, reported cases are highest amongst ages 20–29 years. However, the prevalence of chlamydia and the distribution among the wider population of Belgium is currently unknown.

It is expected, that reported cases likely overrepresent young women, whereas men and older age groups might be underrepresented. Estimation of prevalence and the distribution according to age and gender is essential for the evidence-based design of prevention and control programs, and important for the understanding of sexual transmission dynamics. Therefore, the aim of this study was to estimate the prevalence of genital *C. trachomatis* infections in the population of Belgium aged 16–59 years and to identify relevant risk factors.

## Methods

### CT1 study design and population

**Fig. 1:**
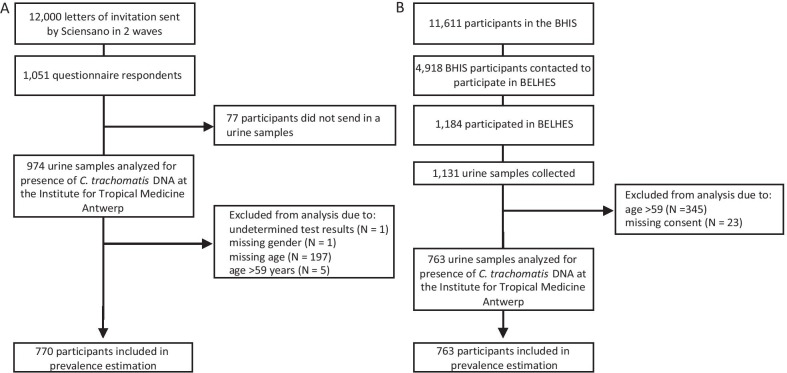
Flowcharts of recruitment, sample collection and data analysis. Two cross-sectional studies **A** CT1 and **B** CT2 were performed to determine the prevalence of genital *Chlamydia trachomatis* infection in the general Belgian population aged 16-59 years. *BHIS* Belgian Health Interview Survey, *BELHES* Belgian Health Examination Survey

The CT1 study followed a cross-sectional design to determine the point-prevalence of chlamydia infection for the Belgian population (11,431,406 inhabitants on 1st January 2019 [14]) and was conducted between 2019–2020. All Belgian residents aged 16–59 years were eligible. The sample size was estimated based on a maximum point-prevalence of 4%, based on studies from other European countries, taking into account an expected response rate of 12%. A randomised representative nationwide sample of 12,000 individuals was drawn from the national register and participation was solicited by mailed letters in two waves. Each wave accounted for 6000 invitations. Representativeness was based on age, nationality, sex and region of residence. No financial incentives were offered, but participants were able to obtain their test results. Informed consent was obtained either through an online survey or mailed letter. Sequence of study procedures are illustrated in Fig. 1A.

### CT1 data and sample collection

After informed consent, participants received a personalized link to the online study questionnaire on the Sciensano hosted LimeSurvey platform or a paper-based questionnaire (see Additional file 1). Questionnaires were available in English, French or Dutch, according to the preference expressed by the participant. Questions focused on sociodemographic variables and sexual behavior as potential risk factors. A maximum of three reminders to complete the questionnaire were sent 1 month after registration. Additionally, participants received a Colli-Pee™ device (Novosanis, Wijnegem, Belgium) sampling kit and were asked to collect a first-void urine sample at home, which was then sent to the laboratory for analysis through regular prepaid mail.

### CT2 study design and population

The CT2 study used urine samples collected through the cross-sectional general Belgian Health Examination Survey (BELHES) in 2018, which was organized as a second stage of the Belgian Health Interview Survey (BHIS) that targeted all Belgian residents without age restriction [15, 16]. Households and respondents within households were selected through a multistage geographically stratified clustered sampling procedure, based on entries from the national register. During the fieldwork of the BHIS, participants aged 18 years and older were invited to participate in a heath examination until a predefined number was reached. No financial incentives were offered, but a free check of cholesterolaemia and glycaemia was available to participants. In total 1184 people participated in the BELHES. The distribution by age, gender and region matched to a large extent the one of the population aged 18 years and older in the BHIS. Sequence of study procedures are illustrated in Fig. 1B. Full details of the methods of both BHIS and BELHES have been described elsewhere [15, 16].

### CT2 data and sample collection

Information on sociodemographic variables and sexual behavior as potential risk factors was obtained through the BHIS study questionnaire (see Additional file 2). Data collection was undertaken during face-to-face interviews at the participant’s home, as well as through a self-administered questionnaire (for the participants > 15 years old) covering sexual behavior, amongst other more sensitive topics. The use of urine samples to trace for chlamydia infection was not included in the initial BELHES examinations. Only urine samples from participants who consented for further use were included in the chlamydia prevalence study.

### Sample processing and analysis

All urine samples were shipped to the National Reference Centre for Sexually Transmitted Infections at the Institute of Tropical Medicine in Antwerp (ITM) for analysis. Samples were tested for the presence of *C. trachomatis* DNA using either the GeneXpert CT/NG Xpert assay (Cepheid, Sunnyvale, California, USA) or the Abbott Real-Time CT/NG assay (Abbott m2000sp and the Abbott m2000rt system, Abbott Molecular Inc., Des Plaines, IL, USA). Both diagnostic methods were validated and accredited by the laboratory at ITM. The results were transferred to Sciensano for linkage to the sociodemographic and behavioral data, and further analysis.

### Study weights

To adjust the prevalence estimation to be representative of the Belgian population, the weight of each participant was calculated. In the CT1 study, participants' weights were calculated based on the percentage of responses divided by percentage of invitations sent, per gender and age group. Only those two variables could be used to calculate the weights, due to privacy and access constraints regarding non-responder data for the initial invited study population.

In the CT2 study, post-stratification weights were applied according to the weight calculated in the BHIS. Those weighting factors were meant to reflect the differential selection probability, correct for differential response rates and adjust for the demographic and geographic sample distribution. Weights were calculated based on cross classified data from the national register on age, gender, household size, quarter of the year in which the interview was conducted and province of residence. Additionally, the selection probability of an individual in the household (1 if household size ≤ 4, < 1 if household size > 4) was taken into account.

### Age groups

For analysis purposes, participants were divided in three age groups: (i) 16–29 years of age in CT1 or 18–29 years of age in CT2, representing the age group with highest reported case numbers by the Belgian laboratory-network surveillance, (ii) 30–44 years of age, representing the population in their reproductive age, (iii) 45–59 years of age, representing the older population, often not included in other prevalence studies.

### Availability of data on the general Belgian population

Statistical data on the age, gender, civil status and nationality of the general Belgian population in 2018 and 2019 were obtained from the open data of Statbel, the Belgian statistical office [14, 17]. Aggregated annual data on the education level was available for the Belgian population > 15 years of age [18].

### Data and statistical analysis

All data analysis was performed using R (version 4.0.3) in R Studio (version 1.3.1093). The crude estimated prevalence was calculated as percentage of participants with *C. trachomatis* positive urine sample in the corresponding study population, by gender and age group. The weighted estimated prevalence and 95% confidence interval (95% CI) was calculated by inclusion of each participant's study weight, as well as household as a cluster variable in CT2, using the survey package (version 4.0). Differences in proportions between groups were compared by two proportions z-test using the prop.test function with Yates continuity correction in the stats package (version 4.0.3). Univariate analysis was performed using the epi.2by2 function with method = “case.control” in the epiR package (version 1.0–15) to calculate the odds ratios (ORs) for possible risk factors associated with chlamydia infection, or non-consent for further use of urine samples in CT2. Multivariable logistic regression analysis was performed using the generalized linear model (glm) function with the logit link in the stats package. Statistical differences in mean age distribution between study groups were calculated using the Wilcoxon rank sum test in the stats package. Correlation between number and type of sexual partners was assessed using the cor function in the stats package with method = “Pearson test”.

## Results

### Study populations

#### CT1

Out of 12,000 individuals invited by letter, 8.8% (1051/12,000) responded to the questionnaire, and 8.1% (974/12,000) submitted a urine sample for analysis (Fig. 1A). Five participants aged 60 years, as well as those with missing information on age or gender, or with undetermined laboratory results (20.9%, 204/974) were excluded. Study population characteristics are presented in Table 1. Among the final study population of 770 participants, 55% (422/770) were women and 45% (348/770) men. Furthermore, 24% (181/770) were aged 16–29 years, 36% (279/770) aged 30–44 years and 40% (310/770) aged 45–59 years. Of note, 59% (456/770) of participants had achieved a high level of education in academia or elsewhere, which was significantly more than in the general Belgian population aged 16–59 years in 2019 (33%, p < 0.0001, Additional file 3: Table S1). Moreover, the proportion of female versus male participants was higher (55% versus 45%, p < 0.01), participants of ages 16–29 years were underrepresented (24% versus 30%, p < 0.001) and of 45–59 years were overrepresented (40% versus 37%, p = 0.03) in CT1, compared to the general population.

**Table 1:** Study population characteristics of the CT1 and CT2 chlamydia prevalence studies

	CT1 study			CT2 study		
	Total (N = 770)	CT negative (N = 759)	CT positive (N = 11)	Total (N = 763)	CT negative (N = 753)	CT positive (N = 10)
Median age of participants, years (IQR)	40 (30, 51)	40 (30, 51)	31 (30, 39)	43 (33, 50)	43 (33, 50)	44 (30, 52)
Median age women, years (IQR)	40 (30, 50)	40 (30, 50)	32 (30, 42)	42 (33, 50)	42 (33, 50)	45 (36, 51)
Median age men, years (IQR)	41 (30, 52)	41 (30, 52)	30 (27, 31)	44 (34, 51)	44 (35, 51)	41 (32, 48)
Age group						
16–29 y/o—18–29 y/o	181 (24%)	178 (24%)	3 (27%)	125 (16%)	122 (16%)	3 (30%)
30–44 y/o	279 (36%)	274 (36%)	5 (45%)	299 (39%)	297 (39%)	2 (20%)
45–59 y/o	310 (40%)	307 (40%)	3 (27%)	339 (44%)	334 (44%)	5 (50%)
Gender						
Male	348 (45%)	343 (45%)	5 (45%)	351 (46%)	348 (46%)	3 (30%)
Female	422 (55%)	416 (55%)	6 (55%)	412 (54%)	405 (54%)	7 (70%)
Nationality						
Belgian	–	–	–	639 (84%)	630 (84%)	9 (90%)
Other than Belgian	–	–	–	124 (16%)	123 (16%)	1 (10%)
Education						
No diploma or primary education	28 (4%)	28 (4%)	0 (0%)	30 (4%)	30 (4%)	0 (0%)
Lower secondary education	45 (6%)	45 (6%)	0 (0%)	73 (10%)	71 (9%)	2 (20%)
Higher secondary education	175 (23%)	172 (23%)	3 (27%)	225 (29%)	223 (30%)	2 (20%)
Higher education (academic or outside University)	456 (59%)	449 (59%)	7 (64%)	382 (50%)	379 (50%)	3 (30%)
No answer	66 (9%)	65 (9%)	1 (9%)	53 (7%)	50 (7%)	3 (30%)
Health literacy						
Low	–	–	–	25 (3%)	24 (3%)	1 (10%)
Limited	–	–	–	209 (27%)	207 (27%)	2 (20%)
Sufficient	–	–	–	472 (62%)	466 (62%)	6 (60%)
No answer	–	–	–	57 (7%)	56 (7%)	1 (10%)
Civil status						
Single (never married)	–	–	–	220 (29%)	215 (29%)	5 (50%)
Married or legal cohabitation	–	–	–	461 (60%)	460 (61%)	1 (10%)
Divorced (not remarried)	–	–	–	78 (10%)	74 (10%)	4 (40%)
Widowed (not remarried)	–	–	–	4 (1%)	4 (1%)	0 (0%)
Sexual intercourse in the last 12 months						
Yes	635 (82%)	625 (82%)	10 (91%)	600 (79%)	593 (79%)	7 (70%)
No	68 (9%)	67 (9%)	1 (9%)	103 (13%)	101 (13%)	2 (20%)
No answer	62 (8%)	62 (8%)	0 (0%)	60 (8%)	59 (8%)	1 (10%)
Nr. of sexual partner in the last 12 months						
One	532 (69%)	526 (69%)	6 (55%)	541 (71%)	535 (71%)	6 (60%)
Two	38 (5%)	38 (5%)	0 (0%)	25 (3%)	24 (3%)	1 (10%)
Three	34 (4%)	34 (4%)	0 (0%)	21 (3%)	21 (3%)	0 (0%)
Four or more	27 (4%)	24 (3%)	3 (27%)	11 (1%)	11 (1%)	0 (0%)
No answer	136 (18%)	134 (18%)	2 (18%)	165 (22%)	162 (22%)	3 (30%)
The last sexual partner was…						
A steady partner	557 (72%)	552 (73%)	5 (45%)	–	–	–
A casual partner	74 (10%)	70 (9%)	4 (36%)	–	–	–
No answer	136 (18%)	134 (18%)	2 (18%)	–	–	–
Condom use during last intercourse						
No	525 (68%)	518 (68%)	7 (64%)	510 (67%)	503 (67%)	7 (70%)
Yes	–	–	–	88 (12%)	88 (12%)	0 (0%)
Yes, the whole time	65 (8%)	64 (8%)	1 (9%)	–	–	–
Yes, but not the whole time	44 (6%)	43 (6%)	1 (9%)	–	–	–
No answer	136 (18%)	134 (18%)	2 (18%)	165 (22%)	162 (22%)	3 (30%)
First sexual intercourse before age 15						
Yes	33 (5%)	33 (5%)	0 (0%)	32 (4%)	31 (4%)	1 (10%)
No	672 (95%)	661 (95%)	11 (100%)	631 (83%)	623 (83%)	8 (80%)
No answer	65 (8%)	65 (9%)	0 (0%)	100 (13%)	99 (13%)	1 (10%)
Median age at first sexual intercourse, years (IQR)	18 (16, 20)	18 (16, 20)	19 (18, 19)	18 (16, 20)	18 (16, 20)	17 (16, 18)
Ever been tested for any STI						
Yes	278 (36%)	272 (36%)	6 (55%)	–	–	–
No	412 (54%)	408 (54%)	4 (36%)	–	–	–
No answer	60 (8%)	59 (8%)	1 (9%)	–	–	–
Ever been diagnosed with chlamydia						
Yes	27 (4%)	27 (4%)	0 (0%)	–	–	–
No	686 (89%)	675 (89%)	11 (100%)	–	–	–
No answer	57 (7%)	57 (8%)	0 (0%)	–	–	–
Sexual orientation for men						
Men who are attracted to men only/mostly/as much as to women	29 (8%)	29 (8%)	0 (0%)	–	–	–
Testing for HIV in the past 12 months						
Yes	–	–	–	53 (7%)	52 (7%)	1 (10%)
No	–	–	–	655 (86%)	647 (86%)	8 (80%)
No answer	–	–	–	55 (7%)	54 (7%)	1 (10%)
Ever been tested for HIV						
Yes	–	–	–	303 (40%)	300 (40%)	3 (30%)
No	–	–	–	405 (53%)	399 (53%)	6 (60%)
No answer	–	–	–	55 (7%)	54 (7%)	1 (10%)
Testing for other STI than HIV in the past 12 months						
Yes	–	–	–	60 (8%)	59 (8%)	1 (10%)
No	–	–	–	608 (80%)	600 (80%)	8 (80%)
No answer	–	–	–	95 (12%)	94 (12%)	1 (10%)
Ever been tested for other STI than HIV						
Yes	–	–	–	321 (42%)	318 (42%)	3 (30%)
No	–	–	–	347 (45%)	341 (45%)	6 (60%)
No answer	–	–	–	95 (12%)	94 (12%)	1 (10%)

CT: *Chlamydia trachomatis*; IQR: interquartile range; y/o: years of age; STI: sexually transmitted infection; Nr.: number; HIV: human immunodeficiency virus

#### CT2

Among the 11,611 participants of the BHIS study, 4918 were contacted to participate in the BELHES. Among those, 24% (1184/4918) agreed to participate and urine samples were collected from 23% (1131/4918) (Fig. 1B). Participants who were above 59 years old (30.5%, 345/1131) were excluded from CT2. Moreover, 2.9% (23/786) of participants did not consent to further use of their sample (Additional file 4: Table S2). There was no significant difference in giving consent according to gender, age group, nationality, education level, civil status or sexual behavior variables. Although, noteworthy all participants who had sexual intercourse before age 15, had been tested for an STI or HIV in the last 12 months, and all but one participant with more than 1 sexual partner in the past 12 months gave consent for further testing. There was a higher proportion of participants with sufficient health literacy level (62% versus 39%, p < 0.05) and lower proportion with limited health literacy (27% versus 57%, p < 0.01) who gave consent. More precisely, participants who did consent for further use were three times more likely to be of sufficient health literacy level (OR = 2.91, 95% CI 1.23, 6.91, p = 0.01, Additional file 5: Table S3). Furthermore, they were nine times more likely to be residents of the Flanders and Wallonia region (OR = 8.92, 95% CI 3.46, 23, p < 0.001) as compared to being residents of Brussels. When we incorporated health literacy status and region of residence in a multivariable logistic regression analysis adjusted for age and gender, both remained significantly associated with giving consent (adjusted OR = 2.82, 95% CI 1.17, 7.10, p = 0.02 and adjusted OR = 11.4, 95% CI 4.38, 35.2, p < 0.00001) (Additional file 5: Table S3).

The final study population of 763 participants belonged to 545 different household clusters and the majority were married or legally cohabiting (60% (461/763)). Study population characteristics are presented in Table 1. Among participants, 54% (412/763) were women and 46% (351/763) men. Furthermore, 16% (125/763) were aged 18–29 years, 39% (299/763) aged 30–44 years and 44% (339/763) aged 45–59 years. Of note, 50% (382/763) of participants had achieved a high level of education in academia or elsewhere, which was significantly more than in the general Belgian population aged 18–59 years in 2018 (33%, p < 0.0001, Additional file 3: Table S1). The proportion of female versus male participants was higher in CT2 (54% versus 46%, p = 0.02), and young participants of age group 18–29 were underrepresented (16% versus 27%, p < 0.0001), while older age groups were over represented, compared to the general population. Moreover, the proportion of married or legally cohabiting participants was higher (60% versus 41%, p < 0.0001) and of single participants lower (29% versus 48%, p < 0.001), than in the general population.

### Chlamydia prevalence estimation and age distribution

#### CT1

The weighted prevalence of chlamydia in the Belgian population aged 16–59 years was estimated at 1.54% (CI 95% 0.78–3.0): 1.32% (CI 95% 0,52–3.0) in women and 1.75% (CI 95% 0.62–4.0) in men (Table 2). The estimated weighted prevalence by age group was 1.54% (CI 95% 0.38–4.0) in 16–29 year olds, 2.07% (CI 95% 0.69–5.0) in 30–44 year olds and 1.01% (CI 95% 0.24–3.0) in 45–59 year olds. Participants with positive results for chlamydia were significantly younger than participants with negative test result (median age and interquartile range (IQR) 31 (30, 39) versus 40 (30, 51), p < 0.0001, Table 1).

**Table 2:** Prevalence estimates of genital *Chlamydia trachomatis* infection in the Belgian population aged 16–59 years

	CT1 study				CT2 study		
	Total, N (%)	Crude prevalence, N (%)	Weighted^a^ prevalence, % (95% CI)		Total, N (%)	Crude prevalence, N (%)	Weighted^b^ prevalence, % (95% CI)
Total	770	11 (1.43)	1.54 (0.78–3)	Total	763	10 (1.31)	1.76 (0.63–4)
*Gender*				*Gender*			
Male	348 (45%)	5 (1.44)	1.75 (0.62–4)	Male	351 (46%)	3 (0.86)	2.25 (0.45–6)
Female	422 (55%)	6 (1.42)	1.32 (0.52–3)	Female	412 (54%)	7 (1.70)	1.29 (0.39–3)
*Age groups total*				*Age groups total*			
16–29 y/o	181 (24%)	3 (1.66)	1.54 (0.38–4)	18–29 y/o	125 (16%)	3 (2.40)	3.54 (0.35–13)
30–44 y/o	279 (36%)	5 (1.79)	2.07 (0.69–5)	30–44 y/o	299 (39%)	2 (0.67)	1.04 (0.08–4)
45–59 y/o	310 (40%)	3 (0.97)	1.01 (0.24–3)	45–59 y/o	339 (44%)	5 (1.47)	1.56 (0.44–4)
*Age groups men*				*Age groups men*			
16–29 y/o	86 (25%)	2 (2.33)	2.15 (0.34–7)	18–29 y/o	59 (17%)	1 (1.69)	5.57 (0.30–23)
30–44 y/o	114 (33%)	2 (1.75)	2.40 (0.39–7)	30–44 y/o	130 (37%)	1 (0.77)	2.07 (0.11–9)
45–59 y/o	148 (43%)	1 (0.68)	0.81 (0.04–4)	45–59 y/o	162 (46%)	1 (0.62)	0.66 (0.04–3)
*Age groups women*				*Age groups women*			
16–29 y/o	95 (23%)	1 (1.05)	0.91 (0.05–4)	18–29 y/o	66 (16%)	2 (3.03)	1.12 (0.15–4)
30–44 y/o	165 (39%)	3 (1.82)	1.75 (0.43–5)	30–44 y/o	169 (41%)	1 (0.59)	0.16 (0.08–1)
45–59 y/o	162 (38%)	2 (1.23)	1.22 (0.2–4)	45–59 y/o	177 (43%)	4 (2.26)	2.40 (0.53–6)

CT: *Chlamydia trachomatis*; y/o: years of age

^a^Participants' weights were calculated based on the percentage of responses divided by percentage of invitations sent, per gender and age group

^b^Post-stratification weights were calculated based on cross classified data on age, gender and province of residence from the national register. Additionally, the selection probability of an individual in the household (1 if household size ≤ 4, < 1 if household size > 4) was taken into account in the calculation of the weights. Household was included as a cluster variable

In women, the estimated weighted prevalence was 0.91% (CI 95% 0.05–4.0) in those aged 16–29 years, 1.75% (CI 95% 0.43–5.0) in those aged 30–44 years and 1.22% (CI 95% 0.20–4.0) in those aged 45–59 years (Table 2).

In men, the estimated weighted prevalence was 2.15% (CI 95% 0.34–7.0) in those aged 16–29 years 2.40% (CI 95% 0.39–7.0) in those aged 30–44 years and 0.81% (CI 95% 0.04–4.0) in those aged 45–59 years (Table 2).

#### CT2

The weighted prevalence of chlamydia in the Belgian population aged 18–59 years was estimated at 1.76% (CI 95% 0.63–4.0): 1.29% (CI 95% 0,39–3.0) in women and 2.25% (CI 95% 0.45–6.0) in men (Table 2). The estimated weighted prevalence by age groups was 3.54% (CI 95% 0.35–13.0) in 18–29 year olds, 1.04% (CI 95% 0.08–4.0) in 30–44 year olds and 1.56% (CI 95% 0.44–4.0) in 45–59 year olds. Participants with positive results for chlamydia were significantly older than participants with negative test result (median age (IQR) 44 (30, 52) versus 43 (33, 50), p < 0.0001, Table 1).

In women, the estimated weighted prevalence was 1.12% (CI 95% 0.15–4.0) in those aged 18–29 years, 0.16% (CI 95% 0.08–1.0) in those aged 30–44 years and 2.40% (CI 95% 0.53–6.0) in those aged 45–59 years (Table 2).

In men, the estimated weighted prevalence was 5.57% (CI 95% 0.30–23) in those aged 18–29 years, 2.07% (CI 95% 0.11–9.0) in those aged 30–44 years and 0.66% (CI 95% 0.04–3.0) in those aged 45–59 years (Table 2).

### Sociodemographic characteristics and risk factors for genital *C. trachomatis* infection

#### CT1

About half of participants with a positive chlamydia test (55%, 6/11) had been tested for STIs before, but never been diagnosed with chlamydia (Table 1). On the other hand, 4% (27/770) amongst all participants had previously been diagnosed with chlamydia. All but one participant with a positive chlamydia test reported having had sexual intercourse in the past 12 months, predominantly with one partner (55%, 6/11). Participants with a positive chlamydia test were more likely to have had three or more sexual partners in the last 12 months (OR = 4.53, 95% CI 1.10–18.6, p = 0.02), although this association was weakened when we adjusted for gender and age in a multivariable logistic regression (adjusted OR = 1.75, 95% CI 0.95–3, p = 0.05, Table 3). Similarly, it was more likely that chlamydia positive participants' last sexual intercourse was with a casual partner as compared to a steady partner (OR = 6.31, 95% CI 1.66, 24.1, p < 0.01), independent of gender and age (adjusted OR = 4.63, 95% CI 1.06–19, p = 0.03). Overall, there was a significant correlation between the number of sexual partners in the last 12 months and type of partner (Pearson coefficient = 0.64, p < 0.0001) and when adjusted for each other, neither remained significant. The majority of participants with a positive chlamydia test (64%, 7/11) reported that they did not use a condom during last intercourse, similarly to the chlamydia negative population (68%, 518/759, Table 1). Among men, 8% (29/348) reported to be attracted to men only, mostly or as much as to women. None of them was among the chlamydia positive participants. Overall, information on sexual behavior was missing in up to 18%, also in the chlamydia positive participants. No gonorrhoea infections were detected in the study population.

**Table 3:** Analysis of risk factors associated with positive urine test for *Chlamydia trachomatis*

CT1 study								
	Negative urine test	Positive urine test	Univariate OR (95% CI)	p-value	Multivariable OR (95% CI)^a^	p-value	Multivariable OR (95% CI)^b^	p-value
Gender								
Female	416	6	1					
Male	343	5	1.01 (0.31, 3.34)	0.99	1.02 (0.29, 3.40)	0.98	1.63 (0.41, 6.87)	0.49
Age group								
16–29 y/o age	178	3	1					
30–44 y/o age	274	5	1.08 (0.26, 4.59)	0.91	1.09 (0.26, 5.35)	0.91	1.13 (0.23, 6.27)	0.88
45–59 y/o age	307	3	0.58 (0.12, 2.90)	0.50	0.580 (0.30, 3.49)	0.51	0.53 (0.07, 3.46)	0.50
Last sexual partner								
Steady	552	5	1					
Casual	70	4	6.31 (1.66, 24.1)	< 0.01	4.63 (1.06, 19)	0.03	3.17 (0.43, 19.7)	0.23
Nr. of sexual partners in the last 12 months								
1 partner	526	6	1					
3 or more partners	58	3	4.53 (1.10, 18.6)	0.02	1.75 (0.95, 3)	0.05	1.28 (0.58, 2.8)	0.53
Ever been tested for STI								
No	408	4	1					
Yes	272	6	2.25 (0.63, 8.05)	0.20	2.11 (0.59, 8.36)	0.25	1.14 (0.27, 4.92)	0.86
Condom use during last intercourse								
Yes	64	1	1					
No	561	8	0.91 (0.11, 7.41)	0.93	1.33 (0.23, 25.4)	0.79	2.13 (0.33, 42.3)	0.50
Level of education (lowest = 1, highest = 4)			1.52 (0.65, 5.74)	0.43	1.62 (0.66, 6.4)	0.39	1.88 (0.59, 12.1)	0.39

CT2 study								
	Negative urine test	Positive urine test	Univariate OR (95% CI)	p-value	Multivariable OR (95% CI)^a^	p-value	Multivariable OR (95% CI)^c^	p-value
Gender								
Female	405	7	1					
Male	348	3	0.50 (0.13, 1.94)	0.31	0.501 (0.11, 1.82)	0.32	0.46 (0.06, 2.18)	0.36
Age group								
18–29 y/o age	122	3	1					
30–44 y/o age	297	2	0.27 (0.05, 1.66)	0.13	0.266 (0.03, 1.63)	0.15	0.23 (0.01, 1.90)	0.21
45–59 y/o age	334	5	0.61 (0.14, 2.59)	0.50	0.611 (0.15, 3.02)	0.50	0.82 (0.14, 4.78)	0.82
Relationship status								
Married or legally cohabitating	460	5	1					
Single	215	1	10.7 (1.24, 92)	0.01	9.59 (1.28, 195)	0.05	7.3 (0.75, 166)	0.11
Single, divorced or widowed	293	9	14.1 (1.78, 112)	< 0.01	15.2 (2.8, 284)	0.01	13.0 (1.98, 254)	0.02
Nr. of sexual partners in the last 12 months								
1 partner	535	6	1					
2 partners	24	1	3.72 (0.43, 32.1)	0.20	3.21 (0.16, 21.4)	0.30	1.38 (0.07, 9.23)	0.08
Sexual intercourse before age 15								
No	623	8	1					
Yes	31	1	2.51 (0.30, 20.7)	0.38	2.36 (0.12, 13.6)	0.43	2.55 (0.13, 17.1)	0.41
Ever been tested for STI (excl. HIV)								
No	341	6						
Yes	318	3	0.54 (0.13, 2.16)	0.37	0.484 (0.10, 1.86)	0.31	0.58 (0.11, 2.77)	0.49
Tested for STI (excl. HIV) in the last 12 months								
No	600	8						
Yes	59	1	1.27 (0.16, 10.3)	0.82	1.20 (0.06, 6.86)	0.87	1.11 (0.05, 7.4)	0.93
Ever been tested for HIV								
No	399	6	1					
Yes	300	3	0.66 (0.16, 2.68)	0.56	0.649(0.13, 2.49)	0.54	0.69 (0.13, 3.32)	0.65
Tested for HIV in the last 12 months								
No	647	8	1					
Yes	52	1	1.56 (0.19, 12.7)	0.68	1.61 (0.09, 9.19)	0.66	1.37 (0.07, 9.18)	0.78
Health literacy level								
Low/limited	231	3	1					
Sufficient	466	6	0.99 (0.25, 4.00)	0.99	1.05 (0.27, 5.06)	0.94	1.44 (0.3, 10.3)	0.67
Nationality								
Belgian	630	9	1					
Other	123	1	0.57 (0.07, 4.53)	0.59	0.553 (0.03, 2.99)	0.58	–	–
Level of education (lowest = 1, highest = 4)			0.807 (0.34, 2.23)	0.65	0.8 (0.33, 2.2)	0.64	0.74 (0.20, 3.09)	0.66

*OR* odds ratio, *CI* confidence interval, *STI* sexually transmitted infection, *excl*. excluding, *HIV* human immunodeficiency virus, *Nr.* number

^a^Multivariable analysis adjusting for gender and age in years

^b^Multivariable analysis adjusting for gender, age in years, number of sexual partners and type of sexual partner

^c^Multivariable analysis adjusting for gender, age in years, number of sexual partners and civil status

#### CT2

All but two participants with a positive chlamydia test reported having had sexual intercourse in the last 12 months (70%, 7/10), predominantly with one partner (60%, 6/10) (Table 1). One participant with positive chlamydia test had 2 sexual partners in the last 12 months and one participant did not provide an answer. Participants with a positive chlamydia test result were more likely to be single, divorced or widowed and not remarried (OR = 14.1, 95% CI 1.78, 112, p < 0.01), independent of gender and age (adjusted OR = 15.2, 95% CI 2.8–284, p = 0.01, Table 3). When we additionally adjusted for number of sexual partners in our multivariable logistic regression the association of civil status with positive chlamydia test result remained significant (OR = 13.0, 95% CI 1.98, 254, p = 0.02). The majority of participants with a positive chlamydia test (70%, 7/10) reported that they did not use a condom during last intercourse, similarly to the chlamydia negative population (67%, 503/759, Table 1). More than half of them had never been tested for human immunodeficiency virus (HIV) (60%, 6/10) or any other STIs (60%, 6/10). Overall, information on sexual behavior was missing in up to 30%, also in the chlamydia positive participants. No gonorrhoea infections were detected in the study population.

## Discussion

Genital infection with *C. trachomatis* is the most diagnosed STI in Belgium. Current clinical screening may be biased towards young sexually active women, due to the medical implications of chlamydia infection for their reproductive health, thereby leading to an underestimation of the frequency of infection in men and older age groups. Therefore, population-wide prevalence studies are needed to assess the true distribution of infections. Based on the analysis of urine samples, we estimated a weighted prevalence of genital chlamydia of 1.54% for the Belgian population 16–59 years of age and 1.76% for the Belgian population 18–59 years of age, through two cross-sectional nationwide studies. We detected no difference in prevalence by gender, where we observed 1.75–2.25% in men and 1.29–1.32% in women.

Our Belgian estimates are comparable to those of other European countries with similar study design. For example, the prevalence in French individuals aged 18–44 was estimated at 1.4% (95% CI 0.8–2.6) for men, and 1.6% (95% CI 1.0–2.5) for women [19]. Similarly, The National Surveys of Sexual Attitudes and Lifestyles (Natsal) conducted in 2010–2012 in the UK, reported chlamydia prevalence of 1.5% (95% CI 1.1–2.0) in women and 1.1% (95% CI 0.7–1.6) in men, amongst participants aged 16–44 years [20].

International studies focusing on younger adults often found higher prevalence in women, which we didn’t observe in our studies. In the Netherlands, the weighted prevalence of chlamydia in individuals aged 18–34 years was 1.1% (95% CI 0.1–7.2) in men and 5.6% (95% CI 3.3–9.5) in women [21]. In Norway, girls aged 15–20 years were twice as likely to be infected than boys (7.3%, 95% CI 5.3–9.7 versus 3.9%, 95% CI 2.3–6.0) [22]. In Spain, on the other hand, the prevalence in young adults aged 15–24 years was similar in men (4.3%, 95% CI 2.9–7.2) and in women (4%, 95% CI 2.8–6.4) [23].

While most national prevalence studies are focused on participants of age 15–35 years, our results show that older age groups should also be included, to capture the distribution of this STI across all demographics and to inform the evidence base regarding population-level chlamydia control [21–23]. Especially in women, we observed the highest weighted prevalence in the age groups of 30–44 years in CT1 and 45–59 years in CT2. Although less clinically relevant for reproductive health in the older population, there is still an associated risk with untreated chlamydia infections for acquisition and transmission of HIV, as well as for the development of cervical cancer, considering coinfection with human papilloma virus, but also as an independent predictor [24, 25].

We detected a significant association between increased number of sexual partners (> 3 in the past 12 months), casual sexual relationships and civil status and genital chlamydia infection, similar to other national prevalence studies from the Netherlands, France and Norway [19, 21, 22]. Condom use was extremely rare among all study participants, but with our sample size we did not detect an association with chlamydia infection, as seen in the Spanish and Norwegian prevalence studies in young adults [22, 23]. Nevertheless, correct and consistent condom use will certainly reduce the risk imposed by casual sex and frequent partner change, as should be emphasized in prevention campaigns with the goal to reduce transmission [26, 27].

However, the high percentage of chlamydia positive cases with only one sexual partner in the last year (55% in CT1 and 60% in CT2) suggests that not having had recent multiple partners does not eliminate risk for infection. Furthermore, three participants with a positive chlamydia test reported that they did not have sexual intercourse in the last 12 months, which could indicate long-term asymptomatic infections. The majority of infections however are estimated to clear spontaneously within one year, without the need for treatment [28, 29]. In a recent review of evidence, it was stated that little is known about the occurrence of tubal damage and squalene in the course of a natural infection, and that the effect of testing on PID prevention was limited [8]. Moreover, the perception of clinical and public health benefit of active case finding and testing for asymptomatic individuals is shifting, due to the lack of high quality empirical evidence for its impact on chlamydia prevalence. Indeed, the harms of stigmatization through screening programs and opportunistic testing, as well as the threat of antimicrobial resistance in other (STI-related) pathogens due to overtreatment in high-income countries, may outweigh the benefits. These concerns should be central in the development of future control strategies.

The findings of our studies are subject to several limitations. A major limitation was the low response rate in CT1 and small sample size in the BELHES, which was used for CT2. The resulting small sample sizes introduce uncertainty around our prevalence estimates, represented in large confidence intervals. Unfortunately, we were not able to assign population-based weights using factors other than age and gender, due to privacy related constraints pertaining to the non-responders in the targeted study population of CT1. For the same reasons, an analysis of non-responders to assess selection bias in CT1 could not be performed. Selection bias may also have occurred during the recruitment for BHIS, and then secondly for BELHES. It has previously been stated that the BELHES responders were of higher education level than non-responders and suffered from underrepresentation of people in the age group of 18–24 years [15]. Moreover, urine was not collected from participants < 18 years of age in the BELHES, which hampered our direct comparison between CT1 and CT2. The relatively low prevalence of chlamydia infection, its heterogeneous distribution in the population and the small sample size made it difficult to estimate stratified prevalence, identify groups at high risk for infection and perform robust multivariate analysis. Moreover, the proportion of missing data on self-reported sexual behavior was ranging up to 30% for some variables, also among chlamydia positive cases, which additionally hindered our risk analysis and possibly introduced social-desirability bias [30].

Furthermore, the population of both studies was skewed towards higher education level, in comparison with the national education levels of 2018 and 2019, as estimated by Statbel [18]. Moreover, we identified health literacy status as a factor in giving consent for further use of participants' samples. This is important to consider, as low education level was identified as a risk factor for chlamydia infection by the Dutch prevalence study [21].

In both studies, participants under the age of 30 were underrepresented, which could have led us to underestimate the prevalence in the youngest age group, which is most targeted by screening efforts.

The high proportion of married compared to single participants in CT2 could have let us to underestimate the prevalence of chlamydia, which was significantly associated with casual relationships in CT1 and civil status in CT2. This selection bias was likely introduced by the household sampling frame in the BHIS.

Lastly, our prevalence estimates based on urine sampling do not include infections through sexual contact of non-genital sites, such as the rectum and oropharynx, which could also have led to underestimation.

Although CT1 had the unique objective to estimate chlamydia prevalence from the beginning, we obtained a comparable overall prevalence estimate in CT2 from using urine sample collected through the BELHES. This suggests, that it is feasible to connect similar studies, aimed at prevalence estimation of STIs, to regular population health surveys in Belgium in the future, for a more integrated approach and concentration of resources. Other established national surveys, such as the Natsal in the UK and the National Health and Nutrition Examination Survey (NHANES), conducted by the CDC in the United States, are good examples of a successful probability sample surveys, which have provided important evidence on age- and sex-specific population prevalence of *C. trachomatis* and other STIs to guide the development of sexual health programmes [20, 31].

## Conclusion

The overall prevalence of chlamydia in the Belgian population is quite low and comparable to other European countries. While screening results point towards a high burden in young females, our results show that the male population, as well as adults above 35 years of age also need to be considered. The key drivers of uncertainty, as identified in our studies, should be considered to guide future research efforts. Effective case management, including appropriate testing and treatment of symptomatic patients and partner notification, as well as prevention strategies targeting behaviors such as condom use, should lead future strategies for national sexual health agendas.

## Supplementary Information

**Additional file 1. **Study Questionnaire CT1 in English.

**Additional file 2. **Questionnaire BHIS and BELHES in English.

**Additional file 3: Table S1.** Comparison of the general Belgian population in 2018 and 2019, and the study populations of CT1 and CT2.

**Additional file 4: Table S2.** Characteristics of BELHES study participants aged 18-59 years, who did or did not consent for further use of their urine sample.

**Additional file 5: Table S3.** Analysis of factors associated with consent for further use of urine sample among BELHES participants 18-59 years of age.

## Data Availability

The datasets used and/or analysed during the current study are available from the corresponding author on reasonable request.

## Abbreviations

BELHES: Belgian health examination survey
BHIS: Belgian health interview survey
*C. trachomatis*: *Chlamydia trachomatis*
CI: Confidence interval
CT1: *Chlamydia trachomatis* prevalence study 1
CT2: *Chlamydia trachomatis* prevalence study 2
DNA: Deoxyribonucleic acid
HIV: Human immunodeficiency virus
ITM: Institute of Tropical Medicine
IQR: Interquartile range
PID: Pelvic inflammatory disease
OR: Odds ratio
STI: Sexually transmitted infection
