# Supplementary material for: Prevalence estimates of genital Chlamydia trachomatis infection in Belgium: results from two cross-sectional studies
Source: BMC Infect Dis. 2021 Sep 14;21:947. doi: 10.1186/s12879-021-06646-y (PMC8439092; doi:10.1186/s12879-021-06646-y)
Supplement: Supplementary file 1 — Additional file 1. Study Questionnaire CT1 in English. [file 12879_2021_6646_MOESM1_ESM.pdf]

sciensano

Questionnaire – Chlamydia study

The following short list of questions may appear very personal to you. They concern your background and sexual behavior. You can be assured that anything you answer will remain strictly anonymous and confidential.

Preference for online?
Please, find at the right a QR-code that directs you to the online survey. When you prefer to complete the survey online, scan this QR-code with your mobile phone or tablet. Or type the following ULR: https://surveys.wiv-isp.be/index.php/923879

You will be asked for a token, which is in your case your study number to be found on the received documents, starting with 'CT'.
When you prefer to complete the paper version, please ignore this QR-code and link and just continue to complete this paper survey.

When you have completed this questionnaire, please use the little envelope to secure your answers. Next, put the closed envelope together with your urine sample in the bubble wrap envelope. Thank you for your participation!

Please cross the box next to the answer that best fits your situation.

1.1. What is your gender?

☐ Man
☐ Woman
☐ I prefer not to answer

1.2. What is your age in years?

__________ years old.

2.1. Have you ever had sexual intercourse?

☐ Yes
☐ No *(continue to question 2.8)*
☐ I don't know / prefer not to answer

2.2. At what age did you first had sexual intercourse?

__________ years old.

2.3. During the past 12 months, have you had sexual intercourse?

☐ Yes
☐ No *(continue to question 2.8)*
☐ I don't know / prefer not to answer

2.4. During the past 12 months, with how many different partners have you had sexual intercourse?

- ☐ 1 partner
- ☐ 2 partners
- ☐ 3 partners
- ☐ 4 partners or more
- ☐ I don’t know / prefer not to answer

2.5. The last time you had sexual intercourse, it was with …

- ☐ a steady partner
- ☐ a casual partner (with whom I have no steady relationship)
- ☐ I don’t know / prefer not to answer

2.6. The last time you had sexual intercourse, did you use a condom?

- ☐ Yes, during the whole time
- ☐ Yes, but not the whole time
- ☐ No
- ☐ I don’t know / prefer not to answer

2.7. During the past 12 months, have you had sexual intercourse with (a) casual partner(s) while you also had a steady partner?

- ☐ Yes
- ☐ No
- ☐ I don’t know / prefer not to answer

2.8. Who do you feel sexually attracted to?

- ☐ Only to men
- ☐ Mainly to men
- ☐ As much to men as to women
- ☐ Mainly to women
- ☐ Only to women
- ☐ I don’t know / prefer not to answer

3.1. Have you ever been tested for sexually transmitted infections (STIs)?

- ☐ Yes
- ☐ No *(continue to question 3.3)*
- ☐ I don’t know / prefer not to answer *(continue to question 3.3)*

3.2. On that occasion, did you have any symptoms related to STIs?

- ☐ Yes
- ☐ No
- ☐ I don’t know / prefer not to answer

3.3. Have you ever been diagnosed with … ? *Multiple answers are possible*

- ☐ Gonorrhea
- ☐ Chlamydia
- ☐ Syphilis
- ☐ Genital/anal warts
- ☐ Hepatitis A
- ☐ Hepatitis B
- ☐ HIV
- ☐ Other STI: ………………………………………………………………….
- ☐ Never been diagnosed *(continue to question 4.1)*
- ☐ I don’t know / prefer not to answer *(continue to question 4.1)*

3.4. When were you last diagnosed with any STI?

- ☐ Within the last 24 hours
- ☐ Within the last 7 days
- ☐ Within the last 4 weeks
- ☐ Within the last 6 months
- ☐ Within the last 12 months
- ☐ Within the last 5 years
- ☐ More than 5 years ago
- ☐ Not applicable

4.1. Do you have a paid job, at this moment, even if it is temporarily interrupted?

- ☐ Yes, paid job (even temporarily interrupted)
- ☐ No, no paid job *(continue to question 4.3)*
- ☐ I don't know/ prefer not to answer

4.2. Do (did) you work fulltime or part-time?

- ☐ Fulltime (*continue to question 4.4*)
- ☐ Part-time (*continue to question 4.4*)
- ☐ I don’t know (*continue to question 4.4*)
- ☐ No answer (*continue to question 4.4*)

4.3. Which of the following profiles is/are applicable to you?
Multiple answers are possible, please, check all that apply.

- ☐ Unemployed, looking for an employment
- ☐ Sickness or invalidity
- ☐ Studies
- ☐ Retirement
- ☐ I do the housekeeping, without pay
- ☐ I work within the family business
- ☐ Other situation, describe: ……………………………………………………………..
- ☐ I don't know/ prefer not to answer

4.4. Do you go to school, or are you a (part time) daytime student?

- ☐ Yes
- ☐ No *(continue to question 4.6)*
- ☐ I don't know / prefer not to answer *(continue to question 4.6)*

4.5. What is your current branch of studies? *If multiple, choose the highest.*

- ☐ Lower education (primary school)
- ☐ Lower secondary education, secondary education of the 1st or 2nd degree
- ☐ Higher secondary education, secondary education of the 3d degree
- ☐ Post-secondary not-higher education
- ☐ Higher education outside the university - short type (A1), professional bachelor
- ☐ Higher education outside the university - long type, master at a college
- ☐ Academic bachelor (college or university)
- ☐ University, licentiate, engineer or master
- ☐ Doctorate
- ☐ Other branch of studies, specify:……………………………………
- ☐ I don't know/ prefer not to answer

4.6. What is your highest diploma or grade?

- ☐ No diploma
- ☐ Lower education (primary school)
- ☐ Lower secondary education, secondary education of the 1st or 2nd degree
- ☐ Higher secondary education, secondary education of the 3rd degree
- ☐ Post-secondary not-higher education
- ☐ Higher education outside university - short type (A1), professional bachelor
- ☐ Higher education outside the university - long type, master at a college
- ☐ Academic bachelor (college or university)
- ☐ University, licentiate, engineer or master
- ☐ Doctorate with thesis
- ☐ Other diploma, specify:………………………………………………
- ☐ I don't know/ prefer not to answer

4.7. How would you assess your health in general?

- ☐ Very good
- ☐ Good
- ☐ Is going (reasonably)
- ☐ Bad
- ☐ Very bad
- ☐ I don’t know / prefer not to answer

Thank you for your participation!

Please use the provided little envelope to send back the questionnaire. Put the closed envelope together with your urine sample in the bubble wrap envelope. Thank you!
