# Supplementary material for: Prevalence estimates of genital Chlamydia trachomatis infection in Belgium: results from two cross-sectional studies
Source: BMC Infect Dis. 2021 Sep 14;21:947. doi: 10.1186/s12879-021-06646-y (PMC8439092; doi:10.1186/s12879-021-06646-y)
Supplement: Supplementary file 2 — Additional file 2. Questionnaire BHIS and BELHES in English. [file 12879_2021_6646_MOESM2_ESM.pdf]

sciensano

STATBEL Belgium in figures

Health Interview Survey, 2018

Respondent

Name :

Code :

Number : _ - _ _ _ _ - _ _ _ - 0 0 - _ _

Interviewer

Number : _ _ _ _

Date of the survey : D D - M M - 2 0 1 8

How to fill in this questionnaire?

- For most questions, tick/cross the box that best matches your reply (ex.: cross the box in front of "yes" if you have once smoked)

EX.01 **Have you smoked more than just a pack of cigarettes in your entire life?**

[X] 1 Yes

[ ] 2 No

- Sometimes you are expected to write down numbers in open boxes (ex.: "19" in reply to the question hereunder)

EX.02 **How old were you when you started smoking?**

I was | 1 | 9 | years old

- In some cases, according to your answer you will be asked a related sub-question (ex.: if you reply "yes" to EX.03, then you should also answer sub-question a) If yes, how many years… (ex.: fill in "6" if the case)

EX.03 **Have you ever smoked daily?**

[X] 1 Yes

a) If yes, how many years have you smoked daily? | | 6 | year

[ ] 2 No

- In other cases, your answer to a question may lead you to "jump" a series of questions and go directly to one that is placed further in the questionnaire (ex.: if you reply "no" to EX.04, you are directed to the question SH.01 on another page)

EX.04 **Have you ever attempted to quit smoking?**

[ ] 1 Yes, several times

[ ] 2 Yes, once

[X] 3 No ⇨ *Go to SH.01 page 3*

- Unless specified otherwise, tick/cross only one reply-box per question, and pass on to the question that follows directly. Do not pay attention to the small figures on the left of the boxes.

Information

In order to guarantee the correct match with the questionnaire through interview, please indicate hereunder your year of birth, gender and the postal code of your residence:

Year of birth: ☐☐☐☐

You are:
1 ☐ a man
2 ☐ a woman

Postal code: ☐☐☐☐

Perceived health

SH.01 **How is your health in general? Is it …**

1 ☐ Very good
2 ☐ Good
3 ☐ Fair
4 ☐ Bad
5 ☐ Very bad

SH.02 **Do you suffer from (have) any chronic (long-standing) illness or condition (health problem)?**

1 ☐ Yes
2 ☐ No

SH.03 **For the past <u>6 months or more</u> have you been limited in activities people usually do because of a health problem?**

1 ☐ Yes, strongly limited
2 ☐ Yes, limited
3 ☐ No, not limited

Stress and well-being

How have you been feeling the last few weeks?

<u>In the past few weeks…</u>

WB.01 **Have you been able to concentrate on whatever you're doing?**

- ☐ 1 Better than usual
- ☐ 2 Same as usual
- ☐ 3 Less than usual
- ☐ 4 Much less than usual

WB.02 **Have you lost much sleep over worry?**

- ☐ 1 Not at all
- ☐ 2 No more than usual
- ☐ 3 Rather more than usual
- ☐ 4 Much more than usual

WB.03 **Have you felt that you are playing a useful part in things?**

- ☐ 1 More so than usual
- ☐ 2 Same as usual
- ☐ 3 Less useful than usual
- ☐ 4 Much less useful

WB.04 **Have you felt capable of making decisions about things?**

- ☐ 1 More so than usual
- ☐ 2 Same as usual
- ☐ 3 Less so than usual
- ☐ 4 Much less capable

In the past few weeks…

WB.05 **Have you felt constantly under strain?**

- ☐ 1 Not at all
- ☐ 2 No more than usual
- ☐ 3 Rather more than usual
- ☐ 4 Much more than usual

WB.06 **Have you felt you couldn't overcome your difficulties?**

- ☐ 1 Not at all
- ☐ 2 No more than usual
- ☐ 3 Rather more than usual
- ☐ 4 Much more than usual

WB.07 **Have you been able to enjoy your normal day-to-day activities?**

- ☐ 1 More so than usual
- ☐ 2 Same as usual
- ☐ 3 Less so than usual
- ☐ 4 Much less than usual

WB.08 **Have you been able to face up to your problems?**

- ☐ 1 More so than usual
- ☐ 2 Same as usual
- ☐ 3 Less able than usual
- ☐ 4 Much less able

In the past few weeks…

WB.09 **Have you been feeling unhappy or depressed?**

- ☐ 1 Not at all
- ☐ 2 No more than usual
- ☐ 3 Rather more than usual
- ☐ 4 Much more than usual

WB.10 **Have you been loosing confidence in yourself?**

- ☐ 1 Not at all
- ☐ 2 No more than usual
- ☐ 3 Rather more than usual
- ☐ 4 Much more than usual

WB.11 **Have you been thinking of yourself as a worthless person?**

- ☐ 1 Not at all
- ☐ 2 No more than usual
- ☐ 3 Rather more than usual
- ☐ 4 Much more than usual

WB.12 **Have you been feeling reasonably happy, all things considered?**

- ☐ 1 More so than usual
- ☐ 2 About same as usual
- ☐ 3 Less so than usual
- ☐ 4 Much less than usual

WB.13 **Have you felt optimistic about your future?**

- ☐ 1 More so than usual
- ☐ 2 About same as usual
- ☐ 3 Less so than usual
- ☐ 4 Much less than usual

VT.01 **On a scale from 0 to 10, where 0 means "not at all satisfied" and 10 means "completely satisfied", how satisfied do you currently feel with your life as a whole?**

☐ — ☐ — ☐ — ☐ — ☐ — ☐ — ☐ — ☐ — ☐ — ☐ — ☐

0 1 2 3 4 5 6 7 8 9 10

not at all satisfied ... completely satisfied

VT.02 **How much during the past weeks…**

(Tick one box per line)	All of the time	Most of the time	Some of the time	A little of the time	None of the time
01. did you feel full of life?	☐ 1	☐ 2	☐ 3	☐ 4	☐ 5
02. did you have a lot of energy?	☐ 1	☐ 2	☐ 3	☐ 4	☐ 5
03. did you feel worn out?	☐ 1	☐ 2	☐ 3	☐ 4	☐ 5
04. did you feel tired?	☐ 1	☐ 2	☐ 3	☐ 4	☐ 5

Eating behaviours

(Tick one box per line)	Yes	No
**EB.01** Have you recently lost more than 6 kilos in a 3-month period?	☐ 1	☐ 2
**EB.02** Do you worry that you have lost control over how much you eat?	☐ 1	☐ 2
**EB.03** Do you make yourself sick because you feel uncomfortably full?	☐ 1	☐ 2
**EB.04** Do others say you are too thin, while you believe yourself to be too fat?	☐ 1	☐ 2
**EB.05** Would you say that food dominates your life?	☐ 1	☐ 2

Other difficulties

AD.01 Over the <u>last two weeks</u>, have you been bothered by the following problems?

(Tick one box per line)	Not at all	Several days	More than half the days	Nearly every day
01. Feeling nervous, anxious or on edge	☐ 1	☐ 2	☐ 3	☐ 4
02. Not being able to stop or control worrying	☐ 1	☐ 2	☐ 3	☐ 4
03. Worrying too much about different things	☐ 1	☐ 2	☐ 3	☐ 4
04. Trouble relaxing	☐ 1	☐ 2	☐ 3	☐ 4
05. Being so restless that it is hard to sit still	☐ 1	☐ 2	☐ 3	☐ 4
06. Becoming easily annoyed or irritable	☐ 1	☐ 2	☐ 3	☐ 4
07. Feeling afraid as if something awful might happen	☐ 1	☐ 2	☐ 3	☐ 4
08. Little interest or pleasure in doing things	☐ 1	☐ 2	☐ 3	☐ 4
09. Feeling down, depressed, or hopeless	☐ 1	☐ 2	☐ 3	☐ 4
10. Trouble falling or staying asleep, or sleeping too much	☐ 1	☐ 2	☐ 3	☐ 4
11. Feeling tired or having little energy	☐ 1	☐ 2	☐ 3	☐ 4
12. Poor appetite or overeating	☐ 1	☐ 2	☐ 3	☐ 4
13. Feeling bad about yourself — or that you are a failure or have let yourself or your family down	☐ 1	☐ 2	☐ 3	☐ 4
14. Trouble concentrating on things, such as reading the newspaper or watching television	☐ 1	☐ 2	☐ 3	☐ 4
15. Moving or speaking so slowly that other people could have noticed? Or the opposite — being so fidgety or restless that you have been moving around a lot more than usual	☐ 1	☐ 2	☐ 3	☐ 4
16. Thoughts that you would be better off dead or of hurting yourself in some way	☐ 1	☐ 2	☐ 3	☐ 4

SU.01 **Have you ever seriously thought of ending your life?**

- ☐ 1 Yes, several times
- ☐ 2 Yes, once
  - a) If yes, did you have such thoughts in the past 12 months?
    - ☐ 1 Yes
    - ☐ 2 No
- ☐ 3 No, never

SU.02 **Have you ever attempted to commit suicide?**

- ☐ 1 Yes, several times
- ☐ 2 Yes, once
  - a) If yes, did you attempt suicide in the past 12 months?
    - ☐ 1 Yes
    - ☐ 2 No
- ☐ 3 No, never

Social contacts

SO.01 **How would you judge your social contacts?**

- ☐ 1 Really satisfying
- ☐ 2 Rather satisfying
- ☐ 3 Rather unsatisfying
- ☐ 4 Really unsatisfying

SO.02 **In general, how many times do you have contact with relatives, children, friends, …?**

- ☐ 1 At least once a week
- ☐ 2 At least once a month
- ☐ 3 At least 3 or 4 times a year
- ☐ 4 At least once a year
- ☐ 5 Never

SO.03 **How many people are so close to you that you can count on them if you have serious personal problems?**

- [ ] 1 None
- [ ] 2 1 or 2
- [ ] 3 3 to 5
- [ ] 4 6 or more

SO.04 **How much concern do people show in what you are doing?**

- [ ] 1 A lot of concern and interest
- [ ] 2 Some concern and interest
- [ ] 3 Uncertain
- [ ] 4 Little concern and interest
- [ ] 5 No concern and interest

SO.05 **How easy is it to get practical help from neighbours if you should need it?**

- [ ] 1 Very easy
- [ ] 2 Easy
- [ ] 3 Possible
- [ ] 4 Difficult
- [ ] 5 Very difficult

Leisure time physical activities

PA.10 **What describes best your leisure time activities <u>during the last year</u>?**

(Only one answer possible)

- [ ] 1 Hard training and competitive sport more than once a week
- [ ] 2 Jogging and other recreational sports or gardening, 4 hours or more per week
- [ ] 3 Jogging and other recreational sports or gardening, less than 4 hours per week
- [ ] 4 Walking, bicycling or other light activities 4 hours or more a week
- [ ] 5 Walking, bicycling or other light activities less than 4 hours a week
- [ ] 6 Reading, watching TV or other sedentary activities

Alcohol consumption

AL.01 **<u>In the past 12 months</u>, how often have you had an alcoholic drink of any kind (beer, wine, cider, breezers, cocktails, premixes, liquor, spirits, homemade alcohol…)?**

- ☐ 1 Every day or almost
- ☐ 2 5 - 6 days a week
- ☐ 3 3 - 4 days a week
- ☐ 4 1 - 2 days a week

⇨ Go to AL02

- ☐ 5 2 - 3 days in a month
- ☐ 6 Once a month
- ☐ 7 Less than once a month

⇨ Go to AL06 page 12

- ☐ 8 Not in the past 12 months, as I no longer drink alcohol ⇨ Go to AL08 page 13
- ☐ 9 Never, or only a few sips or trials in my whole life ⇨ Go to TA.01 page 14

AL.02 **<u>From Monday to Thursday</u>, on how many of these 4 days do you usually drink alcohol?**

- ☐ 1 On all 4 days
- ☐ 2 On 3 of the 4 days
- ☐ 3 On 2 of the 4 days
- ☐ 4 On 1 of the 4 days
- ☐ 5 On none of the 4 days ⇨ Go to AL04 page 12

AL.03 **<u>From Monday to Thursday</u>, how many drinks do you have on average on such a day when you drink alcohol?**

- ☐ 1 16 or more drinks a day
- ☐ 2 10 - 15 drinks a day
- ☐ 3 6 - 9 drinks a day
- ☐ 4 4 - 5 drinks a day
- ☐ 5 3 drinks a day
- ☐ 6 2 drinks a day
- ☐ 7 1 drink a day
- ☐ 8 I don't drink on weekdays

AL.04 **From Friday to Sunday, on how many of these 3 days do you usually drink alcohol?**

- ☐ 1 On all 3 days
- ☐ 2 On 2 of the 3 days
- ☐ 3 On 1 of the 3 days
- ☐ 4 On none of the 3 days ⇨ *Go to AL06*

AL.05 **From Friday to Sunday, how many drinks do you have on average on such a day when you drink alcohol?**

- ☐ 1 16 or more drinks a day
- ☐ 2 10 - 15 drinks a day
- ☐ 3 6 - 9 drinks a day
- ☐ 4 4 - 5 drinks a day
- ☐ 5 3 drinks a day
- ☐ 6 2 drinks a day
- ☐ 7 1 drink a day
- ☐ 8 I don't drink on weekends

AL.06 **In the past 12 months, how often have you had 6 or more drinks containing alcohol on one occasion?**
For instance, during a party, a meal, an evening out with friends, alone at home,…

- ☐ 1 Every day or almost
- ☐ 2 5 - 6 days a week
- ☐ 3 3 - 4 days a week
- ☐ 4 1 - 2 days a week
- ☐ 5 2 - 3 days in a month
- ☐ 6 Once a month
- ☐ 7 Less than once a month
- ☐ 8 Not in the past 12 months
- ☐ 9 Never in my whole life

(8, 9) ⇨ *Go to AL.08*

AL.07 **How frequently have you had at least 4 drinks (*for women*) or at least 6 drinks (*for men*) in 2 hours?**

1 ☐ Every day or almost
2 ☐ Every week, but not daily
3 ☐ Every month, but not weekly
4 ☐ Less than once a month
5 ☐ Not in the past 12 months
6 ☐ Never in my whole life

AL.08 **Not counting small sips, how old were you when you started drinking alcoholic beverages?**

I was ☐☐ years old

AL.09 **Have you ever felt the need to cut down on your drinking?**

1 ☐ Yes
→ **a) If yes, was it** 1 ☐ in the past 12 months? 2 ☐ more than 12 months ago?
2 ☐ No

AL.10 **Have you ever been critisized concerning your drinking?**

1 ☐ Yes
→ **a) If yes, was it** 1 ☐ in the past 12 months? 2 ☐ more than 12 months ago?
2 ☐ No

AL.11 **Have you ever felt guilty about your drinking?**

1 ☐ Yes
→ **a) If yes, was it** 1 ☐ in the past 12 months? 2 ☐ more than 12 months ago?
2 ☐ No

AL.12 **Have you ever felt the need to take a drink first thing in the morning (eye opener)?**

☐ 1 Yes

a) **If yes, was it**
☐ 1 in the past 12 months?
☐ 2 more than 12 months ago?

☐ 2 No

AL.13 **Have you ever been unable to remember what you did or said because you had been drinking?**

☐ 1 Yes

a) **If yes, was it**
☐ 1 in the past 12 months?
☐ 2 more than 12 months ago?

☐ 2 No

Tobacco consumption

Do not refer to the electronic cigarette.

TA.01 **Have you ever smoked even just one whole cigarette in your life?**

☐ 1 Yes

a) If yes, how old were you when you smoked your first whole cigarette?

[|] years old

☐ 2 No *Go to TP01 page 19*

TA.02 **Have you smoked at least 100 cigarettes in total (about 5 packets) or the equivalent amount of tobacco in your whole life?**

☐ 1 Yes

☐ 2 No *Go to TP01 page 19*

TA.03 **Have you ever, in your lifetime, smoked on a daily basis?**

☐ 1 Yes

☐ 2 No *Go to TA.06 page15*

TA.04 **If yes, how old were you when you started smoking <u>daily (even if you stopped smoking daily since)</u>?**

I was ☐☐ years old

TA.05 **If yes, for how many years in total have you smoked daily?**

(Add all separate periods of daily smoking up to today)

I have smoked daily for ☐☐ years in total

(If it is less than a year, write "0")

TA.06 **Do you smoke <u>nowadays</u>?**

1 ☐	Yes, daily	⇨ *Continue with TA.07*
2 ☐	Yes, occasionally	⇨ *Go to TA12 page18*
3 ☐	No, not at all	⇨ *Go to TA.14 page18*

Daily smokers

These questions are only for respondents who currently smoke every day.
Occasional smokers go to question TA.12 page 17.
Ex-smokers go to question TA.14 page 18.

TA.07 **What quantity do you usually smoke <u>per day</u>?**

BEWARE! Please report the number of items you smoke per day, not the number of packs, nor what you smoke occasionally!

01.	I smoke daily	☐☐	cigarettes (rolled &/or manufactured)
02.	I smoke daily	☐☐	cigars/cigarillos
03.	I smoke daily	☐☐	pipefuls of tobacco
04.	I smoke daily	☐☐	sittings of ookah, nargileh, waterpipes
05.	I smoke daily	☐☐	other (specify): ______________________

TA.08 **When do you smoke your first cigarette (cigar, pipe,…) after waking?**

- 1 ☐ Within 5 minutes after waking
- 2 ☐ Within 6 to 30 minutes after waking
- 3 ☐ Within 31 to 60 minutes after waking
- 4 ☐ More than 60 minutes after waking

TA.09 **Have you ever stopped smoking for 24 hours or more because you were trying to quit?**

- 1 ☐ Yes, several times
- 2 ☐ Yes, once
- 3 ☐ No ➪ *Go to TP01 page 19*

TA.10 **When was the last time you attempted to give up smoking and quit for at least 24 hours?**

- 1 ☐ Less than 6 months ago
- 2 ☐ 6 months ago or longer, but less than 12 months ago
- 3 ☐ 12 months ago or longer

TA.11 **Think about the last time you stopped smoking for 24 hours or more because you were trying to quit. What method(s) (if any) did you use to help you quit?**

(More than 1 answer possible)

- 1 ☐ No particular method or assistance
- 2 ☐ Online/phone service "Tabac Stop"
- 3 ☐ Individual or group counselling with a tabacologist (not my doctor)
- 4 ☐ Consultation with a health professional (not tabacoligist)
- 5 ☐ Use of prescribed medicine (Zyban, Champix, ...)
- 6 ☐ Electronic cigarette (with or without nicotine)
- 7 ☐ Nicotine substitutes (patch, gums, spray, tablets,..)
- 8 ☐ Self-help materials through Internet, leaflets, books...
- 9 ☐ Acupuncture, hypnosis, aromatherapy, kinesiology...

Daily smokers: go to TP.01 page 19 !

Occasional smokers

These questions are for respondents who do not smoke every day.
Daily smokers go to question TP.01 page 19.
Ex-smokers go to question TA.14 page 17.

TA.12 **You reported that you smoke, but not every day. How frequently do you actually smoke?**

- ☐ 1 4 to 6 days per week
- ☐ 2 1 to 3 days per week
- ☐ 3 1 to several days a month, but not weekly
- ☐ 4 1 to several days a year, but not monthly
- ☐ 5 Less than 1 day per year

TA.13 **Do you currently smoke more, less or the same amount as 2 years ago?**

- ☐ 1 I smoke more than 2 years ago
- ☐ 2 I smoke less than 2 years ago
- ☐ 3 I smoke as much as 2 years ago

Occasional smokers: go page TP.01 page 19!

Ex-smokers

These questions are for respondents who have smoked in the past, but no longer smoke currently.

TA.14 **How long is it since you have stopped smoking?**

- ☐ 1 Less than 1 month ago
- ☐ 2 1 month ago or longer, but less than 6 months ago
- ☐ 3 6 months ago or longer, but less than 1 year ago
- ☐ 4 1 year ago or longer, but less than 2 years ago
- ☐ 5 2 years ago or longer, but less than 10 years ago
- ☐ 6 10 years ago or longer
- ☐ 7 Not applicable ⇨ *Go to TP.01 page 19*

TA.15 **What method (if any) did you use to help you quit smoking?**

(More than 1 answer possible)

- ☐ 1 No particular method or assistance
- ☐ 2 Online/phone service "Tabac Stop"
- ☐ 3 Individual or group counselling with a tabacologist (not my doctor)
- ☐ 4 Consultation with a health professional (not tabacoligist)
- ☐ 5 Use of prescribed medicine (Zyban, Champix, ...)
- ☐ 6 Electronic cigarette (with or wothout nicotine)
- ☐ 7 Nicotine substitutes (patch, gums, spray, tablets,..)
- ☐ 8 Self-help materials through Internet, leaflets, books...
- ☐ 9 Acupuncture, hypnosis, aromatherapy, kinesiology...

Exposure to tobacco smoke

Questions for all respondents.

TP.01 **How often are you exposed to the tobacco smoke of others indoors (inside the house where you live (at home), in the car, at work, in public places)?**

Only smoke produced by other people should be taken into account.

- ☐ 1 Every day, 1 hour or more a day
- ☐ 2 Every day, less than 1 hour per day
- ☐ 3 At least once a week (but not every day)
- ☐ 4 Less than once a week
- ☐ 5 Never or almost never ⇨ *Ga go to EC.01*

TP.02 **Where are you generally exposed to the tobacco smoke of others indoors ?**

(Multiple answer possible)

- ☐ 1 At home
- ☐ 2 In the car
- ☐ 3 At work
- ☐ 4 In public places (Bars, restaurants, ...)
- ☐ 5 Other, specify: ____________________

Electronic cigarettes (e-cigarettes)

An electronic cigarette (e-cigarette), or similar devices like e-pipe/e-cigar/e-chicha, are little electronical devices that allow simulating the act of smoking but don't burn tobacco and produce vapor from liquids instead.

The following questions refer to all these electronic 'vaping' devices as "e-cigarettes".

EC.01 **Have you ever tried an e-cigarette, even just a few pufs?**

- ☐ 1 Yes
- ☐ 2 No ⇨ *Go to TP.03 page 21*

EC.02 **Are you currently using e-cigarettes?**

- ☐ 1 Yes, every day
- ☐ 2 Yes, once a week or more, but not daily
- ☐ 3 Yes, once a month or more, but not each week
- ☐ 4 Yes, less than monthly
- ☐ 5 No, not at all ⇨ *Go to TP.03 page 21*

EC.03 **What type of e-cigarette do you use the most?**

- ☐ 1 A disposable e-cigarette (non-rechargeable cig-a-like)
- ☐ 2 An e-cigarette that uses replaceable pre-filled cartridges (rechargeable)
- ☐ 3 An e-cigarette with a tank that you refill with e-liquids (rechargeable)
- ☐ 4 A modular system (own combination of separate parts: batteries, atomizers, etc.) that you refill with e-liquids (rechargeable)
- ☐ 5 A device that heats, rather than burns, real tobacco sticks, so it produces vapor instead of smoke (iQOS-type HNB-cigarettes)
- ☐ 6 Don't know

EC.04 **Curently, the e-cigarettes you use the most are…:**

- ☐ 1 without nicotine
- ☐ 2 with nicotine
- ☐ 3 as many with than without nicotine

EC.05 **For how long have you been using e-cigarettes?**

- ☐ 1 Less than 1 month
- ☐ 2 1 month or longer, but less than 6 months
- ☐ 3 6 months or longer, but less than 1 year
- ☐ 4 1 year or longer, but less than 2 years
- ☐ 5 2 years or longer

EC.06 **Before you first used an e-cigarette, did you smoke tobacco?**

- ☐ 1 Yes
- ☐ 2 No

EC.07 **What are your main reasons for using an e-cigarette or a vaping device?**

- ☐ 1 To quit smoking
- ☐ 2 To cut down (not quit) smoking
- ☐ 3 To avoid returning to smoking
- ☐ 4 To use where smoking is forbidden
- ☐ 5 By curiosity, just wanted to try them
- ☐ 6 Because I enjoy it
- ☐ 7 Because it is less harmful than tobacco
- ☐ 8 Because it is cheaper than tobacco
- ☐ 9 Because it does not harm/bother people around me
- ☐ 10 Other reasons (specify): ________________________________

Exposure to e-cigarette vapour

Questions for all respondents.

TP.03 **How often are you exposed to the vapours of electronic cigarette of others indoors (inside the house where you live (at home), in the car, at work, in public places)?**

Only vapours produced by other people should be taken into account.

- ☐ 1 Every day, 1 hour or more a day
- ☐ 2 Every day, less than 1 hour per day
- ☐ 3 At least once a week (but not every day)
- ☐ 4 Less than once a week
- ☐ 5 Never or almost never ⇨ *Go to ID.01 page 22*

TP.04 **Where are you generally exposed to the e-cigarette vapours of others indoors ?**

(Multiple answer possible)

- ☐ 1 At home
- ☐ 2 In the car
- ☐ 3 At work
- ☐ 4 In public places (Bars, restaurants, ...)
- ☐ 5 Other, specify : ______________________________

Consumption of cannabis

ID.01 **Have you ever taken cannabis (hashish or marijuana, also known as weed, shit, dope...)?**

- ☐ 1 Yes
- ☐ 2 No ⇨ *Go to ID.07 page 24*

ID.02 **How old were you when you took cannabis for the first time?**

I was ⎿__⏊__⏌ years old

ID.03 **During the last 12 months, have you taken cannabis?**

- ☐ 1 Yes
- ☐ 2 No ⇨ *Go to ID.07 page 24*

ID.04 **<u>During the last 12 months,</u>**

(Tick one box per line)

		Very often	Quite often	From time to time	Rarely	Never
01.	have you taken cannabis before midday?	☐ 1	☐ 2	☐ 3	☐ 4	☐ 5
02.	have you taken cannabis when you were alone, by yourself?	☐ 1	☐ 2	☐ 3	☐ 4	☐ 5
03.	have you had memory problems when you took cannabis?	☐ 1	☐ 2	☐ 3	☐ 4	☐ 5
04.	have you run into problems because of your use of cannabis (conflict, fight, accident, bad results in school, inefficient at work,…)	☐ 1	☐ 2	☐ 3	☐ 4	☐ 5
05.	have friends or members of your family told you that you ought to reduce your cannabis use?	☐ 1	☐ 2	☐ 3	☐ 4	☐ 5
06.	have you tried to reduce or stop your cannabis use without succeeding?	☐ 1	☐ 2	☐ 3	☐ 4	☐ 5

ID.05 **<u>During the last 30 days</u>, have you taken cannabis?**

- ☐ 1 Yes
- ☐ 2 No ⇨ *Go to ID.07 page 24*

ID.06 **<u>During the last 30 days</u>, on how many days did you take cannabis?**

- ☐ 1 Every day
- ☐ 2 20-29 days
- ☐ 3 10-19 days
- ☐ 4 4-9 days
- ☐ 5 1-3 days

Consumption of other substances

ID.07 **Which other substances have you used, even if it was just once, and when did you last take them?**

(Tick one box per line)	In the past 30 days	In the past 12 months	More than 12 months ago	Never
01. Cocaine	☐ 1	☐ 2	☐ 3	☐ 4
02. Crack	☐ 1	☐ 2	☐ 3	☐ 4
03. Ecstasy (XTC, MDMA)	☐ 1	☐ 2	☐ 3	☐ 4
04. Amphetamines, speed	☐ 1	☐ 2	☐ 3	☐ 4
05. Methamphetamines	☐ 1	☐ 2	☐ 3	☐ 4
06. Ketamine	☐ 1	☐ 2	☐ 3	☐ 4
07. GHB/GBL	☐ 1	☐ 2	☐ 3	☐ 4
08. Heroin	☐ 1	☐ 2	☐ 3	☐ 4
09. LSD or other hallucinogens (magic mushrooms, psilos, DMT, mescaline, ayahuasca…)	☐ 1	☐ 2	☐ 3	☐ 4
10. Opioids not prescribed for you by a doctor (e.g, fentanyls, buprenorphine, oxycodone, codein, ...)	☐ 1	☐ 2	☐ 3	☐ 4
11. New psychoactive substances, known as "NPS" or "research chemicals": e.g. synthetic cannabis, spice, K2, mephedrone, 4-FA, 25I-NBOMe…	☐ 1	☐ 2	☐ 3	☐ 4
12. Medical psychoactive drugs not prescribed for you by a medical doctor (ex. Valium, Rilatin, Rohypnol, Temesta …)	☐ 1	☐ 2	☐ 3	☐ 4

Gambling and betting

GA.01 **<u>In the past 12 months</u>, have you spent any money on games such as lottery (lotto, keno, scratchcards,…), casino games (slot machines, roulette, dice or card games, …), Bingo, betting on sport events or races... ?**

☐ 1 Yes

☐ 2 No ⇨ *Go to VI.01 page 27*

GA.02 **<u>Not</u> including Internet games, <u>in the past 12 months</u>, how often have you bet or spent money on the following activities on location: in retail shops, cafés, casinos, agencies...?**

Do NOT include Internet games here	Every day	Once a week or more	Once a month or more	Less than once a month	Not in the past 12 months	Never
01. Lottery draw tickets: loto, keno, euromillions, rapido…	☐1	☐2	☐3	☐4	☐5	☐6
02. Instant win or scratch cards: Win-for-life, subito, presto…	☐1	☐2	☐3	☐4	☐5	☐6
03. Bingo in pubs and clubs	☐1	☐2	☐3	☐4	☐5	☐6
04. Playing poker for money	☐1	☐2	☐3	☐4	☐5	☐6
05. Slot machines, jackpot	☐1	☐2	☐3	☐4	☐5	☐6
06. Casino games: roulette, black jack, dice…	☐1	☐2	☐3	☐4	☐5	☐6
07. Betting on (horse-) races	☐1	☐2	☐3	☐4	☐5	☐6
08. Betting on sport games	☐1	☐2	☐3	☐4	☐5	☐6
09. Other games for money	☐1	☐2	☐3	☐4	☐5	☐6

GA.03 **<u>In the past 12 months</u>, how often (if ever) have you bet or spent money on <u>Internet</u> games or gambling activities :**

Only Internet games/gambling	Every day	Once a week or more	Once a month or more	Less than once a month	Not in the past 12 months	Never
01. Online lottery tickets: lotto, keno, euromillions, rapido…	☐1	☐2	☐3	☐4	☐5	☐6
02. Online scratch cards: Win-for-life, Astro, Cash,...	☐1	☐2	☐3	☐4	☐5	☐6
03. Online bingo	☐1	☐2	☐3	☐4	☐5	☐6
04. Online poker	☐1	☐2	☐3	☐4	☐5	☐6
05. Online slot machines, jackpot	☐1	☐2	☐3	☐4	☐5	☐6
06. Online casino games: roulette, black jack, dice…	☐1	☐2	☐3	☐4	☐5	☐6
07. Online (horse-) race bets	☐1	☐2	☐3	☐4	☐5	☐6
08. Online bets on sport games	☐1	☐2	☐3	☐4	☐5	☐6
09. Other online games for money	☐1	☐2	☐3	☐4	☐5	☐6

GA.04 **<u>In the past 12 months</u>, how much money did you spend in average per month on bets, gambling or chance games (not counting possible gains)?**

1 ☐ 40€ per month or more ⇨ Please specify |_|_|_|_| Euros per month

2 ☐ Less than 40€ per month ⇨ *Go to VI.01 page 27*

The following questions are about problematic situations that can stem from gambling activitie.

GA.05 **Thinking about your gambling activities <u>in the past 12 months</u>, how often....**

(Tick one box per line)	Almost always	Most of the time	Sometimes	Never
01. have you bet more than you could really afford to lose?	☐ 1	☐ 2	☐ 3	☐ 4
02. have you needed to gamble with larger amounts of money to get the same feeling of excitement?	☐ 1	☐ 2	☐ 3	☐ 4
03. did you go back another day to try to win back the money you lost when you gambled?	☐ 1	☐ 2	☐ 3	☐ 4
04. have you borrowed money or sold anything to get money to gamble?	☐ 1	☐ 2	☐ 3	☐ 4
05. have you felt that you might have a problem with gambling?	☐ 1	☐ 2	☐ 3	☐ 4
06. have people criticized your betting or told you that you had a gambling problem, regardless of whether or not you thought it was true?	☐ 1	☐ 2	☐ 3	☐ 4
07. has gambling caused you any health problems, including stress or anxiety?	☐ 1	☐ 2	☐ 3	☐ 4
08. has your gambling caused any financial problems for you or your household?	☐ 1	☐ 2	☐ 3	☐ 4
09. have you felt guilty about the way you gamble or what happens when you gamble?	☐ 1	☐ 2	☐ 3	☐ 4

GA.06 **<u>In the past 12 months</u>, have you searched/received any professional help in relation to problems caused by your gambling activities?**

1 ☐ Yes

2 ☐ No

Violence

VI.01 **In the past 12 months, have you been a victim of burglary, robbery, or armed robbery, of verbal or psychological violence (eg. insults , threats, isolation), of physical violence (eg. being pushed, being beaten) or of sexual violence (eg. exhibitionism, rape)?**

☐ 1 Yes

☐ 2 No ⇨ *Go to HI.01 page 29*

VI.02 **What type of violence have you personally experienced in the past 12 months, and where did it take place?**

(More than one answer possible)	At home	At work / school	In a public place/on the public road	Elsewhere
01. Burglary, robbery or armed robbery	☐ 1	☐ 2	☐ 3	☐ 4
**Verbal or psychological violence**				
02. Insults, mockery, humiliations, sarcasm, constant criticism	☐ 1	☐ 2	☐ 3	☐ 4
03. Threats, intimidation, blackmail, stalking, denigration, sexual or racist comments/ harassment	☐ 1	☐ 2	☐ 3	☐ 4
04. Isolation, deprivation of freedom	☐ 1	☐ 2	☐ 3	☐ 4
**Physical violence**				
05. Being knocked down, pushed, shaken...	☐ 1	☐ 2	☐ 3	☐ 4
06. Being hit/beaten, wounded with a weapon, strangled,...	☐ 1	☐ 2	☐ 3	☐ 4
**Sexual violence**				
07. Exhibitionism	☐ 1	☐ 2	☐ 3	☐ 4
08. Sexual assault, forced intercourse, rape	☐ 1	☐ 2	☐ 3	☐ 4
**Other**				
09. Specify: ____________________	☐ 1	☐ 2	☐ 3	☐ 4

If you were victim of one or more above-mentionned acts of violence in the past 12 months, please refer to the worst incident when answering the next questions.

VI.03 **As a result of this act of violence, did you consult or contact with one or more of the following persons, services or institutions?**

(More than one response possible)

- ☐ 1 Family (father, mother, brother, sister,...)
- ☐ 2 Friends
- ☐ 3 Trustee at work or in school, Confidential Doctors Bureau (CDB)
- ☐ 4 Police
- ☐ 5 Medical service (practitioner, hospital,…)
- ☐ 6 Psychologist
- ☐ 7 Law- or juridicial service agency, lawyer, courthouse
- ☐ 8 Victim assistance or support services, youth help services, shelter/safe house
- ☐ 9 Call-centers for assistance (télé-accueil, SOS children, SOS sexual abuse, Center for battered women)
- ☐ 10 Other, specify: ____________________
- ☐ 11 I didn't consulted or contacted anyone

VI.04 **It happens sometimes that people know the offender(s) or the perpetrator(s) of the violent incidents. Was/were the offender(s) or perpetrator(s) of these incidents:**

- ☐ 1 Unknown person
- ☐ 2 Colleague(s)
- ☐ 3 Acquaintance(s)
- ☐ 4 Friend(s)
- ☐ 5 My partner
- ☐ 6 My ex-partner
- ☐ 7 My parent(s)
- ☐ 8 My (step)child(ren)
- ☐ 9 Another member of the family
- ☐ 10 Other, specify: ____________________
- ☐ 11 Don't know
- ☐ 12 I prefer not to answer this question

Knowledge and attitudes towards AIDS

HI.01 According to you, ...

		Yes	No	Don't know
01.	is there a risk of HIV transmission when having sex with only one uninfected partner who has no other partners?	☐ 1	☐ 2	☐ 3
02.	Can a person reduce the risk of getting HIV by using a condom every time they have sex?	☐ 1	☐ 2	☐ 3
03.	Can a healthy-looking person have HIV?	☐ 1	☐ 2	☐ 3
04.	Can a person get HIV by hugging or shaking hands with a person who is infected?	☐ 1	☐ 2	☐ 3
05.	Can a person get HIV by drinking from an infected person's glass?	☐ 1	☐ 2	☐ 3

HI.02 Have you ever been tested for HIV?

- ☐ 1 Yes, less than 1 week ago
- ☐ 2 Yes, more than 1 week ago, but less than 3 months ago
- ☐ 3 Yes, more than 3 months ago, but less than 1 year ago
- ☐ 4 Yes, more than one year ago
- ☐ 5 No, never
- ☐ 6 I don't know

(4, 5, 6) ⇨ *Go to HI.04 page 30*

HI.03 We don't want to know the results, but have you been told the results of this test or have you received them?

- ☐ 1 Yes
- ☐ 2 No

HI.04 **Have you ever been tested for a Sexually Transmitted Disease <u>other</u> than HIV?**

- ☐ 1 Yes, less than 1 week ago
- ☐ 2 Yes, more than 1 week ago, but less than 3 months ago
- ☐ 3 Yes, more than 3 months ago, but less than 1 year ago
- ☐ 4 Yes, more than one year ago
- ☐ 5 No, never
- ☐ 6 I don't know

(options 4–6) ⇨ *Go to RH.01*

HI.05 **We don't want to know the results, but have you been told the results of this test or have you received them?**

- ☐ 1 Yes
- ☐ 2 No

Health and Sexuality

The following questions may appear very personal to you. They concern sexual behavior which is also an important health determinant.

You can be assured that anything you answer will remain strictly anonymous and confidential.

RH.01 **Have you ever had sexual intercourse? Please include vaginal, anal and oral sex.**

- ☐ 1 Yes
- ☐ 2 No ⇨ *Go to HL.01 page 33*

RH.02 **How old were you when you first had sexual intercourse?**

I was |__|__| years

RH.03 **In <u>the past 12 months</u>, have you had sexual intercourse?**

- ☐ 1 Yes
- ☐ 2 No ⇨ *Go to HL.01 page 33*

RH.04 **With how many different partners, have you had sexual intercourse in <u>the last 12 months</u>?**

- ☐ 1 1 partner
- ☐ 2 2 partners
- ☐ 3 3 partners
- ☐ 4 4 or more partners
- ☐ 5 Don't know

RH.05 **Did you use a condom <u>the last time</u> you had sexual intercourse?**

- ☐ 1 Yes
- ☐ 2 No
- ☐ 3 Don't know

RH.06 **During <u>the last 12 months</u>, did you - yourself or your partner(s) - use a contraceptive method (to avoid a pregnancy)?**

- ☐ 1 Yes
- ☐ 2 No
- ☐ 3 Not concerned (pregnant or trying to be, menopausal women, women who have relations with women)

⇨ *Go to HL.01 page 33*

RH.07 **Which contraceptive method(s) did you or your partner(s) use during the last 12 months?**

(Mutiple responses possible)

- ☐ 1 A contraceptive pill
- ☐ 2 A patch
- ☐ 3 An implant
- ☐ 4 Injectable contraceptives (such as Depo-Provera)
- ☐ 5 A vaginal ring (such as NuvaRing)
- ☐ 6 An IUD (intra-uterine device)
- ☐ 7 A morning after pill
- ☐ 8 A diaphragm
- ☐ 9 A spermicide or a contraceptive sponge
- ☐ 10 A male condom
- ☐ 11 A female condom
- ☐ 12 Periodical abstention
- ☐ 13 Withdrawal
- ☐ 14 Sterilization of the woman
- ☐ 15 Sterilization of the man
- ☐ 16 Other method. Which one: ______________________________

Health Literacy

There are many situations in everyday life that are important for our health. Some are difficult while we may find others easier. Please indicate for each of the following tasks how difficult or easy they are for you.

HL.01 On a scale from very easy to very difficult, how easy would you say it is to:

(Een kruisje per lijn)	Very easy	Fairly easy	Fairly difficult	Very difficult
01. judge when you may need to get a second opinion from another doctor?	☐ 1	☐ 2	☐ 3	☐ 4
02. use information the doctor gives you to make decisions about an illness?	☐ 1	☐ 2	☐ 3	☐ 4
03. find information on how to manage certain mental health problems like stress or depression?	☐ 1	☐ 2	☐ 3	☐ 4
04. judge if the information on health risks in the media is reliable? (Examples: TV, Internet or other media)	☐ 1	☐ 2	☐ 3	☐ 4
05. find out about activities that are good for your mental well-being? (Examples: meditation, sport, walking,...)	☐ 1	☐ 2	☐ 3	☐ 4
06. understand information in the media on how to get healthier? (Examples: Internet, newspapers, magazines)	☐ 1	☐ 2	☐ 3	☐ 4

Quality of Life

© EuroQol Research Foundation. EQ-5D™ is a trade mark of the EuroQol Research Foundation.

Belgium (English) v2.0: We are not authorized to publish the questions.

QL.01

QL.02

QL.03

QL.04

QL.05

Thank you for your collaboration!

Do you have any remarks?

Télé-accueil is a (French-speaking) help line or service that offers professional help to people in crisis, in distress or in need of emotional / psychological support

This service is available for callers 24/24h all year round.

Confidentiality and anonymity are warranted.

Télé-accueil

Tel.: **107**

Web: www.tele-accueil.be

Sciensano
Lifestyle and chronic diseases
Juliette Wytsman street, 14
1050 Brussels

Stefaan Demarest	02/642 57 94
Lydia Gisle	02/642 57 53
Rana Charafeddine	02/642 57 39

E-mail : his@sciensano.be

web: www.healthsurvey.be
