# Supplementary material for: Prevalence estimates of genital Chlamydia trachomatis infection in Belgium: results from two cross-sectional studies
Source: BMC Infect Dis. 2021 Sep 14;21:947. doi: 10.1186/s12879-021-06646-y (PMC8439092; doi:10.1186/s12879-021-06646-y)
Supplement: Supplementary file 3 — Additional file 3: Table S1. Comparison of the general Belgian population in 2018 and 2019, and the study populations of CT1 and CT2. [file 12879_2021_6646_MOESM3_ESM.docx]

**Table S1: Comparison of the general Belgian population in 2018 and 2019 and the study populations of CT1 and CT2.**

|  | **Belgian population age 16-59 years old**  **excerpt in 2019***  **(N = 6,498,952)** | **CT1 Total**  **(N = 770)** | **P-value**** | **Belgian population age 18-59 years old**  **excerpt in 2018***  **(N = 6,246,834)** | **CT2 Total**  **(N = 763)** | **P-value**** |
| --- | --- | --- | --- | --- | --- | --- |
| **Gender** |  |  |  |  |  |  |
| Male | 3,273,602 (50%) | 348 (45%) | <0.01 | 3,143,705 (50%) | 351 (46%) | 0.02 |
| Female | 3,225,350 (50%) | 422 (55%) | <0.01 | 3,103,129 (50%) | 412 (54%) | 0.02 |
| **Nationality** |  |  |  |  |  |  |
| Belgian | 5,539,786 (85%) | - |  | 5,333,023 (85%) | 639 (84%) | n.s. |
| Other | 959,166 (15%) | - |  | 913,811 (15%) | 124 (16%) | n.s. |
| **Civil status** |  |  |  |  |  |  |
| Single (never married) | 3,292,042 (51%) | - |  | 2,983,852 (48%) | 220 (29%) | <0.0001 |
| Married or legal cohabitation | 2,496,936 (38%) | - |  | 2,540,753 (41%) | 461 (60%) | <0.0001 |
| Divorced (not remarried) | 655,314 (10%) | - |  | 664,612 (11%) | 78 (10%) | n.s. |
| Widowed (not remarried) | 54,660 (1%) | - |  | 57,617 (1%) | 4 (1%) | n.s. |
| **Study Age Groups** |  |  |  |  |  |  |
| 16-29 / 18-29 y/o | 1,915,003 (30%) | 181 (24%) | <0.001 | 1,666,778 (27%) | 125 (16%) | <0.0001 |
| 30-44 y/o | 2,213,641 (34%) | 279 (36%) | n.s. | 2,203,909 (35%) | 299 (39%) | 0.03 |
| 45-59 y/o | 2,370,308 (37%) | 310 (40%) | 0.03 | 2,376,147 (38%) | 339 (44%) | <0.001 |
| **Education** | **Belgian population >15 years of age 2019*****  **N = 9,387,140** |  |  | **Belgian population >15 years of age 2018*****  **N = 9,330,349** |  |  |
| No diploma or primary education | 1,064,640 (11%) | 28 (4%) | <0.0001 | 1,128,546 (12%) | 30 (4%) | <0.0001 |
| Lower secondary education | 1,854,192 (20%) | 45 (6%) | <0.0001 | 1,843,367 (20%) | 73 (10%) | <0.0001 |
| Higher secondary education | 3,383,883 (36%) | 175 (23%) | <0.0001 | 3,304,576 (35%) | 225 (29%) | <0.001 |
| Higher Education  (academic or outside University) | 3,084,424 (33%) | 456 (59%) | <0.0001 | 3,053,861 (33%) | 382 (50%) | <0.0001 |
| No answer | - | 66 (9%) |  | - | - |  |

* All data were obtained from Statbel (https://statbel.fgov.be/en)

**The study populations of CT1 and CT2 were compared to the general Belgian population by z-test for equality of proportions with Yates continuity correction. P-value applies to both studies unless otherwise indicated. n.s = non significant difference.

*** Unfortunately information on age is not included in the information about education level and therefore the population could not be adjusted to represent the exact same age group as the CT1 or CT2 study population.
