# Supplementary material for: Prevalence estimates of genital Chlamydia trachomatis infection in Belgium: results from two cross-sectional studies
Source: BMC Infect Dis. 2021 Sep 14;21:947. doi: 10.1186/s12879-021-06646-y (PMC8439092; doi:10.1186/s12879-021-06646-y)
Supplement: Supplementary file 4 — Additional file 4: Table S2. Characteristics of BELHES study participants aged 18-59 years, who did or did not consent for further use of their urine sample. [file 12879_2021_6646_MOESM4_ESM.docx]

**Table S2: Characteristics of BELHES study participants aged 18-59 years who did or did not consent for further use of their urine sample.**

|  | **Total**  **(N = 786)** | **No consent**  **(N = 23)** | **Consent for further use**  **(N = 763)** | **P-value*** |
| --- | --- | --- | --- | --- |
| **Median age of participants, years (IQR)** | 43 (33, 50) | 42 (36, 48) | 43 (33, 50) |  |
| **Age group** |  |  |  |  |
| 18-29 y/o | 130 (17%) | 5 (22%) | 125 (16%) | n.s. |
| 30-44 y/o | 309 (39%) | 10 (43%) | 299 (39%) | n.s. |
| 45-59 y/o | 347 (44%) | 8 (35%) | 339 (44%) | n.s. |
| **Gender** |  |  |  |  |
| Male | 362 (46%) | 11 (48%) | 351 (46%) | n.s. |
| Female | 424 (54%) | 12 (52%) | 412 (54%) | n.s. |
| **Nationality** |  |  |  |  |
| Belgium | 657 (84%) | 18 (78%) | 639 (84%) | n.s. |
| Other | 129 (16%) | 5 (22%) | 124 (16%) | n.s. |
| **Education** |  |  |  |  |
| No diploma or primary education | 30 (4%) | 0 (0%) | 30 (4%) | - |
| Lower education | 77 (10%) | 4 (17%) | 73 (10%) | n.s. |
| Higher secondary education | 394 (50%) | 12 (52%) | 382 (50%) | n.s. |
| Higher Education  (academic or outside University) | 229 (29%) | 4 (17%) | 225 (29%) | n.s. |
| No answer | 56 (7%) | 3 (13%) | 53 (7%) | - |
| **Health literacy** |  |  |  |  |
| Low | 25 (3%) | 0 (0%) | 25 (3%) | - |
| Limited | 222 (28%) | 13 (57%) | 209 (27%) | <0.01 |
| Sufficient | 481 (61%) | 9 (39%) | 472 (62%) | <0.05 |
| No answer | 58 (7%) | 1 (4%) | 57 (7%) | - |
| **Civil status** |  |  |  |  |
| Single (never married) | 227 (29%) | 7 (30%) | 220 (29%) | n.s. |
| Married or legal cohabitation | 475 (60%) | 14 (61%) | 461 (60%) | n.s. |
| Divorced (not remarried) | 80 (10%) | 2 (9%) | 78 (10%) | n.s. |
| Widowed (not remarried) | 4 (1%) | 0 (0%) | 4 (1%) | - |
| **Sexual intercourse in the last 12 months** |  |  |  |  |
| Yes | 619 (79%) | 19 (83%) | 600 (79%) | n.s. |
| No | 105 (13%) | 2 (9%) | 103 (13%) | n.s. |
| No answer | 62 (8%) | 2 (9%) | 60 (8%) | n.s. |
| **Nr. of sexual partner in the last 12 months** |  |  |  |  |
| One | 559 (71%) | 18 (78%) | 541 (71%) | n.s. |
| Two | 25 (3%) | 0 (0%) | 25 (3%) | - |
| Three | 21 (3%) | 0 (0%) | 21 (3%) | - |
| Four or more | 12 (2%) | 1 (4%) | 11 (1%) | n.s. |
| No answer | 169 (22%) | 4 (17%) | 165 (22%) | - |
| **Condom use during last intercourse** |  |  |  |  |
| Yes | 92 (12%) | 4 (17%) | 88 (12%) | n.s. |
| No | 525 (67%) | 15 (65%) | 510 (67%) | n.s. |
| No answer | 169 (22%) | 4 (17%) | 165 (22%) | - |
| **First sexual intercourse before age 15** |  |  |  |  |
| Yes | 32 (4%) | 0 (0%) | 32 (4%) | - |
| No | 652 (83%) | 21 (91%) | 631 (83%) | n.s. |
| No answer | 102 (13%) | 2 (9%) | 100 (13%) | - |
| **Median age at first sexual intercourse, years (IQR)** | 18 (16, 20) | 17 (16, 18) | 18 (16, 20)) |  |
| **Testing for HIV in the past 12 months** |  |  |  |  |
| Yes | 53 (7%) | 0 (0%) | 53 (7%) | - |
| No | 677 (86%) | 22 (96%) | 655 (86%) | n.s. |
| No answer | 56 (7%) | 1 (4%) | 55 (7%) | - |
| **Ever been tested for HIV** |  |  |  |  |
| Yes | 314 (40%) | 11 (48%) | 303 (40%) | n.s. |
| No | 416 (53%) | 11 (48%) | 405 (53%) | n.s. |
| No answer | 56 (7%) | 1 (4%) | 55 (7%) | - |
| **Testing for other STI than HIV in the past 12 months** |  |  |  |  |
| Yes | 60 (8%) | 0 (0%) | 60 (8%) | - |
| No | 629 (80%) | 21 (91%) | 608 (80%) | n.s. |
| No answer | 97 (12%) | 2 (9%) | 95 (12%) | n.s. |
| **Ever been tested for other STI than HIV** |  |  |  |  |
| Yes | 333 (42%) | 12 (52%) | 321 (42%) | n.s. |
| No | 356 (45%) | 9 (39%) | 347 (45%) | n.s. |
| No answer | 97 (12%) | 2 (9%) | 95 (12%) | - |
| **Region of residence** |  |  |  |  |
| Brussels | 201 (26%) | 17 (74%) | 184 (24%) | 2.592e-07 |
| Flanders | 351 (45%) | 0 (0%) | 351 (46%) | n.s. |
| Wallonia | 234 (30%) | 6 (26%) | 228 (30%) | n.s. |

*** Proportions between participants who consented and who did not consent were compared using z-test for equality of proportions with Yates continuity correction. n.s = non significant difference.

*Abbreviations: CT= Chlamydia trachomatis, IQR = interquartile range, y/o = years of age , STI = sexually transmitted infection, Nr. = number, HIV = human immunodeficiency virus*
