# Supplementary material for: Prevalence estimates of genital Chlamydia trachomatis infection in Belgium: results from two cross-sectional studies
Source: BMC Infect Dis. 2021 Sep 14;21:947. doi: 10.1186/s12879-021-06646-y (PMC8439092; doi:10.1186/s12879-021-06646-y)
Supplement: Supplementary file 5 — Additional file 5: Table S3. Analysis of factors associated with consent for further use of urine sample among BELHES participants 18-59 years of age. [file 12879_2021_6646_MOESM5_ESM.docx]

**Table S3: Analysis of factors associated with non-consent for further use of urine sample among BELHES participants 18-59 years of age.**

|  | **No consent** | **Consent for further use** | **OR (95% CI)** | **P-value** | **Adj. OR* (95% CI)** | **P-value** |
| --- | --- | --- | --- | --- | --- | --- |
| **Gender** |  |  |  |  |  |  |
| Female | 12 | 412 | 1 |  |  |  |
| Male | 11 | 351 | 0.93 (0.41, 2.13) | 0.86 |  |  |
| **Nationality** |  |  |  |  |  |  |
| Belgian | 18 | 639 | 1 |  |  |  |
| Other | 5 | 124 | 0.70 (0.25, 1.92) | 0.48 |  |  |
| **Education** |  |  |  |  |  |  |
| No/Low education | 4 | 103 | 1 |  |  |  |
| Higher education | 16 | 607 | 1.47 (0.48, 4.49) | 0.49 |  |  |
| **Health literacy level** |  |  |  |  |  |  |
| Low/limited | 13 | 234 | 1 |  |  |  |
| Sufficient | 9 | 472 | 2.91 (1.23, 6.91) | 0.01 | 2.82 (1.17-7.10) | 0.02 |
| **Relationship status** |  |  |  |  |  |  |
| Married or legally cohabitating | 14 | 461 | 1 |  |  |  |
| Single | 9 | 302 | 1.02 (0.44, 2.38) | 0.97 |  |  |
| **Ever been tested for STI (excl. HIV)** |  |  |  |  |  |  |
| No | 9 | 341 | 1 |  |  |  |
| Yes | 12 | 321 | 0.69 (0.29, 1.67) | 0.41 |  |  |
| **Ever been tested for HIV** |  |  |  |  |  |  |
| No | 11 | 405 | 1 |  |  |  |
| Yes | 11 | 303 | 0.75 (0.32, 1.75) | 0.50 |  |  |
| **Number of sexual partners in the last 12 months** |  |  |  |  |  |  |
| 1 partner | 18 | 541 | 1 |  |  |  |
| >1 partner | 1 | 57 | 1.90 (0.25, 14.5) | 0.53 |  |  |
| **Condom use during last intercourse** |  |  |  |  |  |  |
| No | 15 | 510 | 1 |  |  |  |
| Yes | 4 | 88 | 0.65 (0.21, 1.99) | 0.45 |  |  |
| **Belgian region** |  |  |  |  |  |  |
| Brussels | 17 | 184 | 1 |  |  |  |
| Flanders/Wallonia | 6 | 579 | 8.92 (3.46, 23) | <0.001 | 11.4 (4.38-35.2) | <0.00001 |

** = multivariable logistic regression adjusted for gender, age in years, health literacy level and region.*

*Abbreviations: OR = odds ratio, CI = confidence interval, STI = sexually transmitted infection, HIV = human immunodeficiency virus, adj. = adjusted.*
